# A Solution‐Based Deposition Method Enabling Pigment Blue Edible Electrochemical Transistors

**DOI:** 10.1002/advs.202416141

**Published:** 2025-02-27

**Authors:** Alessandro Luzio, Fabrizio M. Ferrarese, Matteo Butti, Alberto D. Scaccabarozzi, Bojan Petrović, Sanja Kojić, Goran Stojanović, Simone Fiorini Granieri, Shubham Tanwar, Adrica Kyndiah, Mario Caironi

**Affiliations:** ^1^ Center for Nano Science and Technology Istituto Italiano di Tecnologia Via Rubattino, 81 Milano 20134 Italy; ^2^ Department of Physics Politecnico di Milano Piazza Leonardo da Vinci, 32 Milano 20133 Italy; ^3^ Faculty of Medicine University of Novi Sad Hajduk Veljkova 3 Novi Sad 21000 Serbia; ^4^ Faculty of Technical Sciences University of Novi Sad T. Dositeja Obradovića 6 Novi Sad 21000 Serbia

**Keywords:** copper phthalocyanine, edible electronics, edible semiconductor, OMIEC, organic transistor

## Abstract

Copper(II) phthalocyanine (CuPc), also known as Pigment Blue 15, is a widely utilized pigment renowned for its exceptional semiconducting properties when refined to electronic‐grade purity. Recent studies have confirmed its safety if ingested at doses required for essential active components in edible electronics for advanced gastrointestinal tract monitoring. Since in‐body operations impose stringent safety constraints on operational biases, the development of transistors with high transconductance at low voltages is required to ensure adequate amplification gain. This study presents a simple and cost‐effective method for producing solution‐processed CuPc films characterized by a unique porous microstructure that facilitates efficient volumetric ion uptake and mixed ionic‐electronic conductivity in electrolyte‐gated devices. These porous films exhibit capacitance 30 times greater than compact CuPc films produced through conventional physical vapor deposition methods. The resulting edible transistors demonstrate *On/Off* ratios exceeding 10^3^ and channel width‐normalized transconductance of up to 50 µS mm^−1^ at 0.8 V, establishing their potential as critical active components in future edible devices. Moreover, the proposed method results in a limited impact of impurities on CuPc charge transport efficiency, thus affecting the purification costs and, crucially, enabling the sourcing of CuPc pigments through recycling and upcycling.

## Graphical abstract

A novel solution‐based method is presented to produce Copper Phthalocyanine fibrous films displaying effective ion permeability and enhanced transconductance in electrolyte‐gated transistors architectures. Using these films, the first instance of a fully edible electrolyte‐gated transistor with volumetric capacitance, targeting in‐body applications, is demonstrated. Additionally, the effective sourcing of CuPc is showcased from the upcycling of Pigment Blue 15.

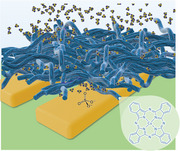

## 1 Introduction

Electronics is becoming increasingly pervasive and profoundly impacting daily life, spanning industries such as healthcare, communication, and leisure. However, pursuing enhanced functionality in electronic systems often comes at the expense of environmental sustainability and biocompatibility. Edible Electronics, an interdisciplinary research field merging electronics, materials science, food science, and biology, promises a revolutionary leap in developing biocompatible and eco‐friendly sensor technologies. At the forefront of sustainable development, edible electronic devices are envisioned to emulate the natural fate of food within the gastrointestinal tract, where some components are metabolized, and others are safely eliminated through fecal excretion.^[^ ^1^ ^]^ These groundbreaking devices may open novel opportunities in real‐time health monitoring, drug delivery, and biofeedback systems, potentially transforming healthcare services and our understanding of physiological well‐being. Additionally, unprecedented applications in the food sector can be envisaged, facilitating food quality monitoring and waste reduction. A recognized materials design strategy for this field, which has already been embraced in recent research endeavors, entails the use of food and established food additives as the sole functional constituents for all the device components. This principle is exemplified by recently published edible conductive pastes and inks exploiting the conductive properties of edible activated carbon (E153),^[^ ^2^, ^3^ ^]^ as passive sensors designed for applications in the food industry,^[^ ^4^ ^]^ healthcare,^[^ ^5^ ^]^ and groundbreaking edible robotics.^[^ ^6^, ^7^ ^]^ An unprecedented leap forward in the development of edible electronic active components, capable of acquiring information and generating/modulating signals within the gastrointestinal tract, is exemplified by the first instances of entirely edible and nutritive power sources, including supercapacitors^[^ ^8^ ^]^ and rechargeable batteries.^[^ ^9^ ^]^ Simultaneously, a variety of substrates, electronic conductors, and ionic conductors have been studied and recognized as tangible candidates for constructing the first fully edible integrated circuits.^[^ ^10^, ^11^ ^]^ In this context, a notable limitation of this compelling research domain consists in an insufficient library of effective edible semiconductors, to be employed as the active material in logic and synaptic circuits, and sensing and biosensing platforms. Many food colorants and non‐toxic or even nutritive natural dyes, such as carotenoids, flavonoids, anthocyanins, and certain vitamins, possess an extended π‐conjugation and an electronic structure resembling those of the acknowledged organic semiconductors.^[^ ^1^, ^12^, ^13^ ^]^ Nevertheless, they are predominantly affected by structural and chemical constraints that hinder charge transport within thin film nanostructures. The carotenoids family stands out as a notable exception, having already been successfully employed in organic field‐effect transistors (OFETs).^[^ ^14^, ^15^, ^16^, ^17^, ^18^ ^]^ Nevertheless, their transport properties remain insufficient, and they are critically susceptible to chemical instability when exposed to a combination of light and atmospheric oxygen.^[^ ^19^, ^20^ ^]^ Alternatively to food colorants, several pigments commercially employed in cosmetics and food‐contact applications may be considered due to their established biocompatibility and limited bioavailability. A notable example is Copper(II) Phthalocyanine (CuPc), which stands out as one of the most widely utilized and cost‐effective blue colorants (with the commercial name of Pigment Blue 15) for many industrial sectors, such as outdoor painting and coating, packaging, textile, plastics, and a variety of cosmetics.^[^ ^21^ ^]^ Upon purification to *electronic‐grade*, it has been largely adopted as a semiconductor for optoelectronic devices, such as OFETs, Light‐Emitting Diodes, and organic photovoltaic cells.^[^ ^22^, ^23^, ^24^, ^25^, ^26^, ^27^, ^28^ ^]^ Its incorporation in well‐recognized toothpaste formulations as a whitening agent over the past 15 years without evidence of eventual toxicity is an indirect indication of its oral safety. Through a critical review of pertinent clinical trials and a simulation of CuPc ingestion via toothbrushing, Feltri et al. recently established that at least 1 mg per day of CuPc can be safely ingested, based on a minimum of two toothbrushing sessions per day. This finding ultimately validates the use of CuPc as a semiconductor for edible electronics experimentation.^[^ ^29^, ^30^, ^31^ ^]^ Notably, by finely tuning the parameters of the Physical Vapor Deposition process, which is currently the most widely recognized deposition method for this pigment,^[^ ^23^, ^32^ ^]^ Feltri et al. have accomplished the first demonstration of a completely edible chitosan‐based Electrolyte Gated Organic Field‐Effect Transistor (EGOFET), despite the restricted selection of processes, materials, and treatments imposed by edibility criteria. Nevertheless, in‐body applications impose strict safety constraints on operational biases, setting the range of device voltage operating conditions well below 1.5 V according to IEC 60601‐1, which is the international benchmark establishing the general safety and performance requirements for medical electrical equipment,^[^ ^33^ ^]^ to prevent interference with the normal electrophysiological processes of the human body. This creates a need for edible transistors with transconductance values significantly higher than those of standard EGOFETs at low applied voltages, well below 1 V. Higher transconductances can be obtained in transistors characterized by a volumetric capacitance, i.e., a capacitance that increases with the volume of the semiconducting film, as in the case of Organic Electrochemical Transistors (OECTs). In such an operating regime, the gating capacitance largely surpasses the ideal electrical double‐layer value of typical EGOFETs (generally below 10 µF cm^−^^2^ in terms of equivalent areal capacitance). OECTs may improve signal amplification and enhance the sensitivity of sensing systems.^[^ ^34^ ^]^ They also may allow efficient voltage operation at low biases in logic gates^[^ ^35^ ^]^ and provide synaptic‐like plasticity for neuromorphic signal propagation and circuits.^[^ ^36^ ^]^ As a result, OECTs hold promise as crucial components for future edible electronics, including sensors, biosensors, and communication devices.

In this study, we introduce a novel solution‐based approach that utilizes an intermediate molecular protonation step^[^ ^37^ ^]^ to fabricate CuPc films that exhibit volumetric capacitance, typical of OECTs, and good charge transport properties. Such films are characterized by a porous microstructure that enables efficient ion uptake when interfaced with an electrolyte gating medium. By utilizing impedance analysis and OECT test platforms, we demonstrate volume‐dependent capacitances exceeding 140 µF cm^−^^2^ and transconductance normalized to the channel width reaching many tens of µS mm^−1^. Importantly, we demonstrate limited susceptibility to the purity level of CuPc with respect to previous literature. The latter is a key aspect to allow the use of low‐cost and even recycled pigment sources, as we exemplify by fabricating efficient transistors based on CuPc directly extracted from a toothpaste formulation. Finally, through the integration of solution‐processed CuPc films into edible transistor structures,^[^ ^11^ ^]^ we mark the first generation of edible OECTs. These devices demonstrate *On/Off* current ratios surpassing 10^3^ and transconductance levels up to 50 µS mm^−1^, holding promise as components in highly sensitive edible biosensors and low‐voltage driven edible integrated circuits. The expectation of reduced costs, resulting from the exclusive utilization of solution‐based methods and the maintenance of low raw material expenses, point to the realistic implementation of disposable electronic systems for advanced applications, such as Point of Care Testing (POCT) and supply chain food monitoring.

## 2 Solution Processing and Transferring of CuPc Films

**Figure 1:**
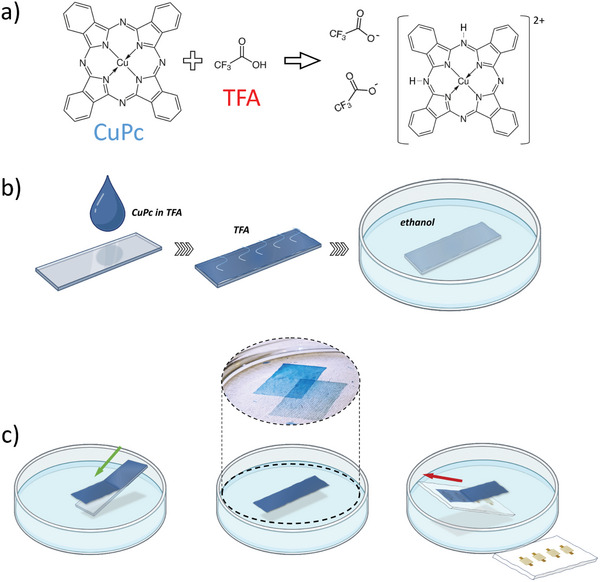
a) CuPc and TFA chemical structures and TFA‐driven CuPc protonation and solvation mechanism; b) scheme of CuPc films processing, including drop casting from TFA solution, wet film drying and residual TFA removal by ethanol bathing; c) transfer process of CuPc film: delamination in water and recollection with a water‐insoluble substrate. A real picture of the CuPc film floating in water is also reported.

Extensive investigation into the solubility of CuPc has led to the exclusion of the majority of common organic solvents, with the only exception of Trifluoroacetic acid (TFA).^[^ ^38^ ^]^ TFA, a strong halogenated derivative of acetic acid, is generally utilized in the acid‐catalyzed synthesis of peptides and proteins,^[^ ^39^ ^]^ and has previously been employed as a solubilizing agent for CuPc, mainly in the context of electrophoretic deposition,^[^ ^40^, ^41^, ^42^ ^]^ and just sporadically in association with solution coating techniques.^[^ ^43^, ^44^, ^45^ ^]^ In **Figure** **1**a, the distinctive solvation mechanism of CuPc in TFA is elucidated. This mechanism involves the creation of a molecular complex through the protonation of the peripheral aza nitrogen atoms of CuPc, enabling the coordination of protonated CuPc with TFA^−^ ions. Specifically, in line with established literature and consistent with the absorption spectrum of CuPc dissolved in TFA (Figure S1, Supporting Information), a trans di‐proton adduct (CuPcH_2_^+^) appears to be generated.^[^ ^37^ ^]^ However, the precise determination of the number of protons coordinating each CuPc molecule in a pure TFA solution has not been definitively established and may potentially exceed a value of 2.^[^ ^40^, ^42^ ^]^ Through the above mentioned solvation mechanism, CuPc solubility up to 2·10^−3^ mol kg^−1^ (1.7 g L^−1^) can be finally obtained.^[^ ^38^ ^]^

When a TFA solution of CuPc is deposited on standard glass substrates by drop‐casting (depicted in Figure 1b), highly uniform wet films are readily formed. This outcome can be attributed to the acidic nature of the solvent, which fosters a chemical interaction with the substrate, likely involving a superficial ionization process.^[^ ^43^, ^46^ ^]^ This interaction promotes the spreading and the even distribution of the solution on the glass surface. Upon the subsequent drying of the wet films, the complete coating of the substrates, up to ∼20 cm^2^ areas, is realized. By simply adjusting parameters such as drop volume, ink concentration, and substrate size, films with controlled mass per unit area can be obtained (in this work, in a range from 7.5 µg cm^−2^ up to 75 µg cm^−2^). Successively, a post‐processing step, aimed at effectively eliminating the toxic TFA retained within the film, must be implemented. This objective can be equivalently achieved through two alternative steps: i) a high‐temperature thermal annealing process (exceeding 150 °C);^[^ ^43^ ^]^ or ii) by immersing the films in a non‐toxic solvent bath (such as ethanol bath), capable of dissolving and removing the residual TFA without adversely affecting the chemical and structural integrity of the CuPc films (Figure 1b).

The development of edible electronic systems necessitates the compatibility of the involved deposition processes with edible substrates, which typically have limited resistance to solvents, leading to dissolution or swelling, and heat exposure.^[^ ^11^ ^]^ This is the case for all cellulose derivatives, which are bio‐derived materials of considerable interest in the field of edible electronics. These materials offer ease of processing and notable physical properties, including lightweight, transparency, flexibility, and mechanical strength.^[^ ^47^, ^48^, ^49^ ^]^ Among these, ethyl cellulose, a food additive designated as E462, stands out due to its insolubility in water, a feature that makes it especially suitable for biosensing applications and operation in physiological environments.^[^ ^50^, ^51^, ^52^ ^]^ Regrettably, its solubility in most organic solvents and alcohols (such as TFA and ethanol, utilized in the dissolution of CuPc and the film cleaning stage), coupled with its relatively low softening point (in the 100 °C ÷ 130 °C range), prohibits its use as a substrate for CuPc solution processing. A general approach to circumvent these compatibility issues involves decoupling the semiconductor film formation process from its incorporation into edible devices. To achieve this objective, we have implemented a Floating Film Transfer Method, which is recognized as a convenient strategy for producing self‐standing organic electronic devices with enhanced conformability.^[^ ^53^, ^54^ ^]^ Recent advancements, including the formation of 2D crystals through “solution epitaxy” and the (static/dynamic) Floating Transfer of polymeric films,^[^ ^55^, ^56^, ^57^, ^58^, ^59^ ^]^ have demonstrated the feasibility of transferring free‐standing, nanometric semiconducting films from a liquid surface to arbitrary, large area substrates, or into multilayered devices such as heterojunctions, to avoid the dissolution of the underlayers.^[^ ^60^, ^61^, ^62^ ^]^

The successive steps of our transferring process are depicted in Figure 1b,c: first, the TFA solution is drop casted onto a hydrophilic carrier substrate, such as glass (Figure 1b); second, the film is let to dry, and the residual TFA is removed via ethanol bath (Figure 1b); third, the CuPc film is detached from the glass surface by allowing it to float on water (Figure 1c); fourth, the CuPc film is collected from water using a non‐water‐soluble support, such as the edible ethyl cellulose substrates here adopted (Figure 1c).

In our process, thoroughly cleaned and oxygen plasma etched microscope glass slides were utilized as temporary supports for the solution casting of CuPc. Importantly, during the “floating off” stage, a distinct separation of the CuPc films from the glass substrate occurs upon initial contact with water, without the need for sacrificial interlayers. This phenomenon can be attributed to the differential hydrophilicity between bare glass and CuPc, which promotes water/glass interaction while repelling water from CuPc. As evidenced by the picture reported in Figure 1c, this floating step does not compromise the integrity of CuPc films. Floating occurs independently from the mass per unit area of the film, ultimately underscoring the robustness of the small molecule polycrystalline film. We successfully reproduced the entire process—from drop‐casting the CuPc solution to transferring the CuPc film—on areas as large as 7 × 7 cm^2^ (Figure S1, Supporting Information), clearly indicating strong compatibility with large‐area substrates, especially considering that the entire procedure is currently performed manually, without any automation, process control, or parameter optimization.^[^ ^63^ ^]^

## 3 Optical and Microstructural Characterization of TFA‐Processed CuPc Films

**Figure 2:**
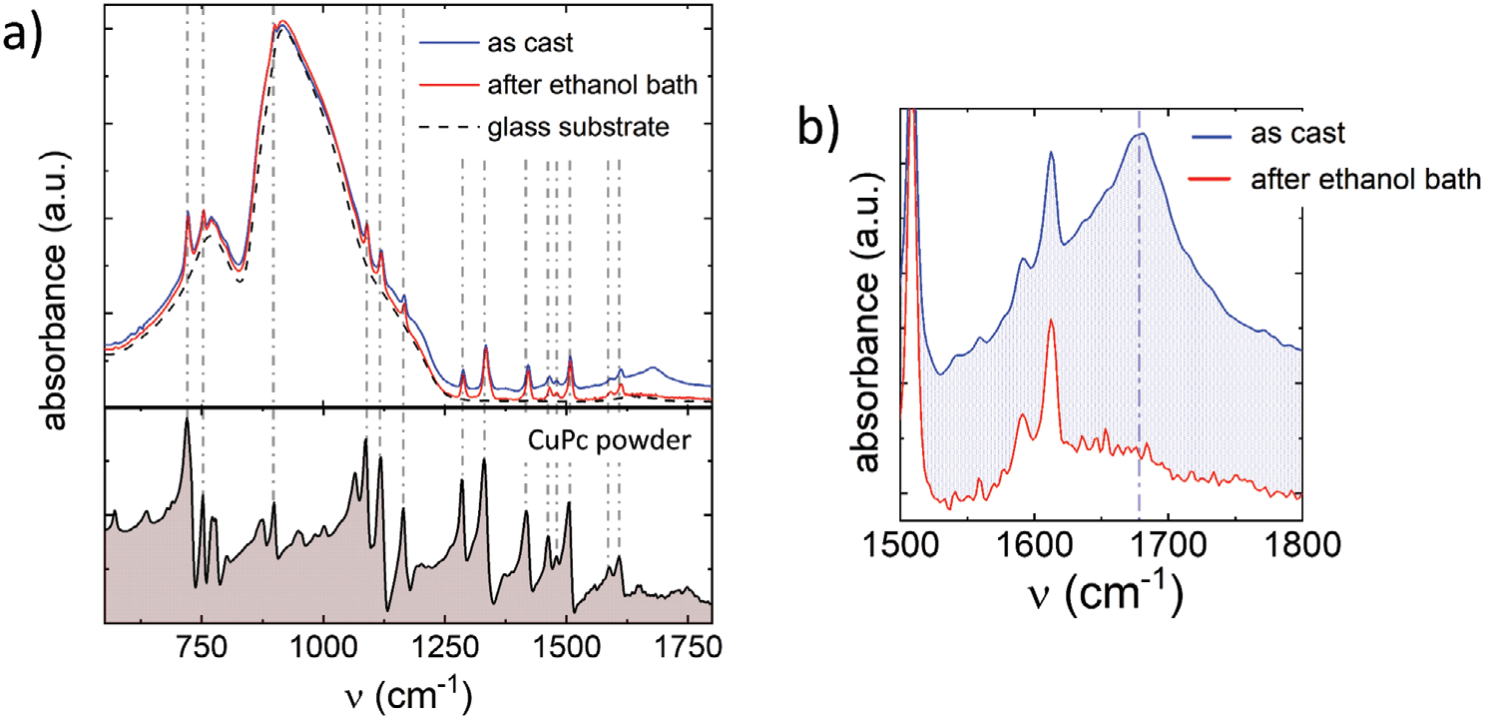
a) FTIR spectra of CuPc films, as cast (blue line) and after the ethanol bath (red line). Dashed gray lines refer to the absorption peaks in the CuPc unprocessed powder spectrum (black, full line); b) magnification of FTIR spectra in the 1500 cm^−1^ ÷ 1800 cm^−1^ range.

To assess the chemical integrity and the actual composition of CuPc films after TFA processing, Fourier‐Transform Infrared Spectroscopy (FTIR) analysis was conducted on both as cast films and those subjected to an ethanol bath, as reported in **Figure** **2**a. Both spectra exhibited quasi‐identical peaks pattern, superimposed on the signal arising from the glass substrate supporting the CuPc films. Remarkably, the same peaks pattern is observed in the unprocessed powder, implying that TFA does not alter the vibrational modes of CuPc, i.e., its chemical structure is retained, despite the intermediate protonation step (Figure 1a). Nevertheless, two additional features, not present in the powder spectrum, are distinctly observed in the spectrum of as‐cast films: a peak at approximately ~1670 cm^−1^ and a broad band spanning the high frequencies range (Figure 2b). The first peak is typically associated with the vibrational modes of carboxyl moieties and is frequently linked to acetic acid derivatives, such as TFA, denoting the presence of residual TFA within the as‐cast films.^[^ ^64^, ^65^ ^]^ The second contribution was previously associated with charge transfer phenomena and ascribed to a doping effect of TFA on CuPc.^[^ ^43^ ^]^ Importantly, both these contributions disappear following the ethanol bath, unequivocally indicating the successful removal of all residual TFA molecules.

**Figure 3:**
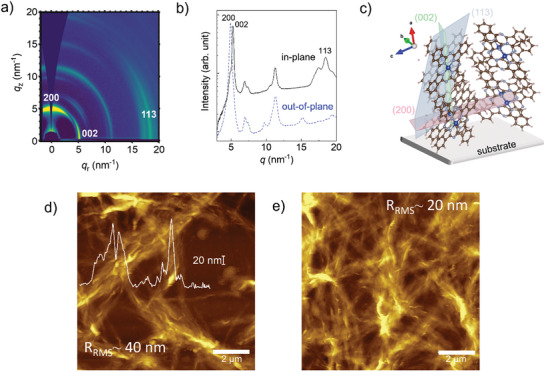
a) Representative 2D‐GIWAXS pattern of solution‐processed CuPc films and b) corresponding profiles along the out‐of‐plane (dashed blue line) and in‐plane (solid black line) directions. c) Representation of the molecular arrangements; d) AFM topography of a CuPc film with 15 µg mm^−2^ mass per unit area; d) AFM topography of a CuPc film with 30 µg mm^−2^ mass per unit area.

To elucidate the structural properties of solution processed CuPc films with variable thickness and investigate their correlation with different processing and purification conditions, we performed Grazing Incidence Wide Angle X‐Ray Scattering (GIWAXS), reported in **Figure** **3**. GIWAXS profiles of all films show qualitatively similar patterns (Figure 3a), exhibiting multiple intense diffraction peaks both in the low‐*q* and high‐*q* regions, suggesting a high degree of molecular order. Corresponding profiles along the in‐plane and out‐of‐plane directions are shown in Figure 3b. In agreement with the reported literature on CuPc packing motifs, our GIWAXS patterns are identified by the alpha phase polymorph, as also suggested by UV‐vis data (see Figure S2, Supporting Information), displaying the (200) crystal direction mostly out‐of‐plane, while the (002) and (113) are mostly in‐plane. This means that CuPc molecules stand mostly upright, facing each other parallel to the substrate, as illustrated in Figure 3c, with a herringbone molecular arrangement, as already reported.^[^ ^66^, ^67^ ^]^ Alongside the alpha phase, GIWAXS patterns reveal additional minor diffraction maxima that cannot be attributed to this molecular arrangement (Figure S3, Supporting Information). These diffractions are probably associated to the coordination of residual TFA^−^ with protonated CuPc that leads to the formation of a new crystal family, similarly to what has been reported for other doped molecules,^[^ ^68^ ^]^ supporting the evidence of a superior polymorphic diversity.^[^ ^69^ ^]^ Importantly, the ethanol bath leads to the suppression of the extra diffractions and to the recovery of a neat CuPc α‐phase microstructure, in strong agreement with our FTIR spectra, confirming residual TFA removal and deprotonation of CuPc upon ethanol washing.

AFM measurements were conducted on solution‐processed CuPc films, as reported in Figures 3d,e and Figure S4 (Supporting Information). In all films, elongated domains, ultimately forming a fibrous‐like film texture, are observed. When a small amount of CuPc is deposited, with a mass per unit area ≤7.5 µg cm^−^^2^, the fibers uniformly cover the substrate, forming a compact layer with a thickness ≤50 nm and a low roughness (*R_RMS_* ≈4.3 nm, Figure S4b,c, Supporting Information). As the mass per unit area increases, the fiber‐like domains spontaneously organize into larger fibrillar bundles, each with a thickness of no less than 120 to 140 nm (Figure 3d). This leads to a highly porous, woven microstructure with R_RMS_ roughness values ranging from ≈40 to ≈20 nm, depending on the bundle density (Figure 3d,e; Figure S3d–g, Supporting Information). Importantly, following the float‐off and recollection process with ethyl cellulose substrates, the film microstructure remains intact (Figure S3h,i, Supporting Information).

## 4 Electrical Characterization of TFA‐Processed CuPc Films

**Figure 4:**
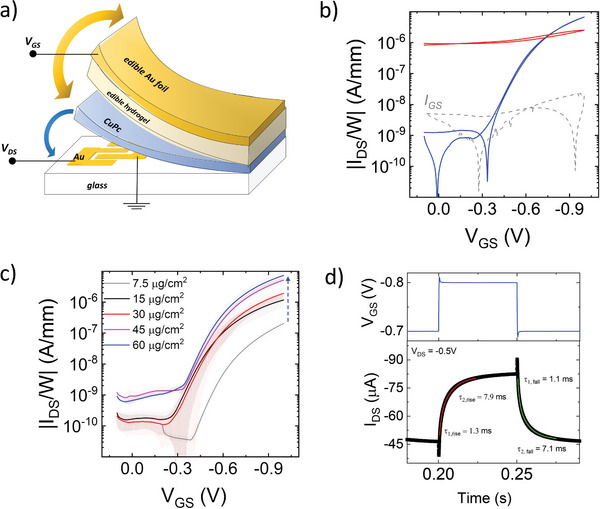
Scheme of the CuPc‐based OECTs on glass a); the edible, laminable, and removable hydrogel consists of an *agar‐agar* layer imbibed with water and edible salts (for the devices in Figure Na_2_SO_4_ 0.1 m water solution); representative transfer characteristic curves of 60 µg cm^−2^ thick CuPc OECTs, as cast (red) and after the ethanol bath (blue) b); mean transfer characteristic curves and point by point standard deviation (over 10 measurements on different OECTs) of CuPc based OECTs with variable mass per area c); transient response of the drain current *I_DS_* when the gate is pulsed from *V_GS_* = −0.7 V to *V_GS_* = −0.8 V, at *V_DS_* = −0.5 V for a duration of 50 ms. The red and green line represents the exponential fit of the τ_rise_ and τ_fall_ respectively d).

The transport properties of solution‐processed CuPc films were preliminary assessed using solid‐state, bottom‐gate and top‐gate OFETs as test‐bench devices. A detailed description of the OFET structures employed and their current‐voltage *I*–*V* characteristic curves are reported in the Figure S5 (Supporting Information). Strong *p‐*type current modulation is observed, exhibiting field‐effect mobility (*µ*) values typically exceeding 10^−3^ cm^2^ V^−1 ^s^−1^, and reaching up to 10^−2^ cm^2^ V^−1^ s^−1^, with *On/Off* ratios superior to 10^4^. These values compare favorably with those obtained using conventional vacuum deposition techniques, which yield *µ* values approaching 10^−2^ cm^2^ V^−1^ s^−1^ only under specific conditions, such as deposition onto heated substrates (up to 180 °C)^[^ ^70^ ^]^ and/or highly engineered surfaces.^[^ ^23^, ^30^ ^]^ Additionally, these values are very sporadically surpassed in literature, limitedly to epitaxially grown CuPc films (10^−1^ cm^2^ V^−1^ s^−1^)^[^ ^71^ ^]^ and to single crystals (≈1 cm^2^ V^−1^ s^−1^).^[^ ^26^ ^]^ To test the CuPc performances as the active phase in Electrolyte‐Gated Transistors, we have employed the test‐bench architecture depicted in **Figure** **4**a: CuPc films were transferred onto glass substrates with photolithographically defined gold Source and Drain patterns, following the procedure depicted in Figure 1c. These devices were completed by manually placing a layer of an edible hydrogel on top of the CuPc surface to work as the electrolytic gating medium, namely an agar‐agar polysaccharide film (E406), embedding edible salts water solutions. Finally, a fragment of commercial gold foil (E175), with an area exceeding 1 cm^2^ and mechanically placed on top of the hydrogel, was used as the Gate electrode. We further evaluated CuPc in electrolyte‐gated transistors using various biocompatible electrolyte systems, demonstrating strong compatibility across a broad spectrum of biocompatible environments. Representative transfer characteristic curves are presented in Figure S6 (Supporting Information).

In Figure 4b and Figure S7 (Supporting Information), the transfer curves of OECTs with 60 µg cm^−2^ CuPc are reported before and after the TFA removal. The transistor was measured in the saturation regime (drain voltage *V_DS_* = −0.5 V); in all plots, Source to Drain current values *I_DS_* were normalized to the transistor channel width (*I_DS_*/*W*). It can be observed that devices with as‐cast CuPc films are normally *On*, with constant *I_DS_*/*W* ≈1 µA mm^−1^ in the gate voltage (*V*_GS_) range between 0.1 and −0.6 V, followed by a small charge modulation, up to 2 × from *V*_GS_ = −0.6 to −1 V. Such behavior can be attributed to CuPc doping, induced by residual TFA in the film after solution casting,^[^ ^43^ ^]^ as revealed by FTIR and GIWAXS data on as‐cast CuPc films. After the ethanol bath, almost four orders of magnitude of *p‐*type current modulation are gained in the same device when sweeping *V*_GS_ from 0.1 to −1 V, denoting the effective solvent/dopant removal. More in detail, in the *Off* state (*V_GS_* = 0 V), *I_DS_*/*W* is negligible, and the current flow through the device is predominantly governed by the leakage through the hydrogel gating media (*I_GS_*/*W* ≈5 nA mm^−1^). Conversely, in the *On* state (*V_GS_* = −1 V), we measured *I_DS_*/W ≈7 µA mm^−1^, leading to an *On/Off* current ratio ≈10^3^.

The transfer characteristics of electrolyte‐gated transistors with variable CuPc mass per unit area are reported in Figure 4c. All the curves exhibit *p‐*type current modulation over 3 to 4 orders of magnitude when sweeping the gate voltage (*V*_GS_) from 0.1 to −1 V. It can be observed that *I_DS_*/*W* is strongly influenced by the mass per unit area, indicating a volumetric gating effect and therefore an OECT behavior (Figure S8, Supporting Information). Specifically, in the case of 7.5 µg cm^−2^ (Figure 3d), we measured a mean *I_DS_*/*W* of 0.2 µA mm^−1^ (with a standard deviation of 32%) in full channel accumulation (*V*_GS_ = −1 V), in good agreement with recently reported values of hydrogel‐gated transistor based on evaporated CuPc.^[^ ^30^ ^]^ Increasing the mass per area to 15 µg cm^−2^, corresponding to the initial formation of fiber bundles within the CuPc microstructure (Figure 3e), results in a 6× increase of *I_DS_*/*W*, up to 1.2 µA mm^−1^ (with a standard deviation of 50%) at *V_GS_* = −1 V.

From 15 to 60 µg cm^−^^2^, *I_DS_*/W continues to increase, albeit more gradually, reaching ≈7.3 µA mm^−1^ (with a standard deviation of 30%). Beyond 60 µg cm^−^^2^, an opposite trend and a subsequent decrease in the *On* currents is observed (Figure S9, Supporting Information).

A key aspect of the electrolyte‐gated transistors is their transient behavior, which directly influences signal response time in biosensing and neuromorphic applications, as well as the switching speed and power consumption of circuits. To investigate the transient response of the transistor with 60 µg cm^−2^ CuPc, we applied a voltage pulse as input to the gate *V*_GS_, and the output drain current *I*_DS_ is recorded as a function of time. Figure 4e depicts the time response of the transistor when we pulse the gate from *V_GS_* = −0.7 V to *V_GS_* = −0.8 V for a duration of 50 ms, while the *V_DS_* = −0.5 V. The response times τ_rise/fall_ were obtained by fitting the I_DS_ (t) versus time with a biexponential equation $I_{ds}\;\left(t\right)=A_{1}\;e^{-(\frac{t-t_{0}}{\tau_{1(rise/fall)\;}})}+A_{2}e^{-(\frac{t-t_{0}}{\tau 2\;(rise/fall)})},$ where τ_1(rise/fall)_ corresponds to the initial rise or decay due to the input voltage pulse and τ_2(rise/fall)_ is correlated to slower processes likely due to structural reorganization or ion/electronic charge reorganization^[^ ^72^ ^]^ and A1 and A2 are constants. Specifically, the extracted times are τ_1,rise_ = 1.3 ms, τ_2,rise_ = 7.9 ms and τ_1_,_fall_ = 1.1 ms, τ_2_,_fall_ = 7.1 ms. For application in biosensing such as electrophysiological monitoring, conservatively setting the critical time response at τ_2_, we can observe that it falls in the same range as the state‐of‐the‐art OECTs, thus suggesting that the device is suitable for these applications.^[^ ^73^, ^74^ ^]^

**Figure 5:**
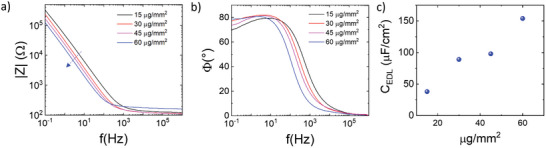
Bode plots (impedance modulus a) and phase b)) of EIS measurements on porous and compact CuPc films with variable thickness interfaced with agar hydrogels; electrical double layer capacitance values C_EDL_ (extracted assuming the Randles circuital model) versus mass per unit area.

**Figure 6:**
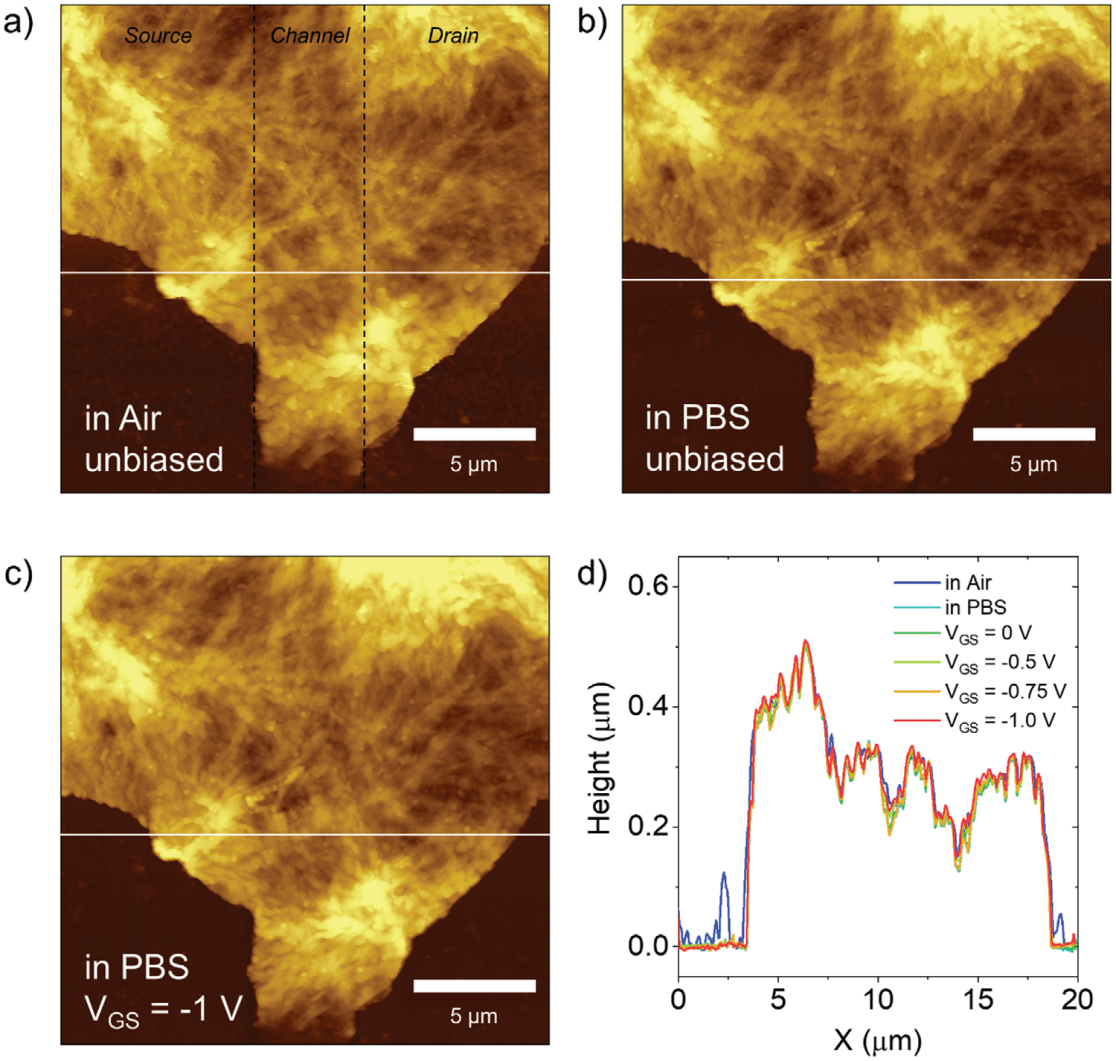
In Situ AFM Measurements of a CuPc film. AFM topography a) in Air unbiased, b) in PBS solution unbiased, and c) in PBS solution with applied V_GS_ = −1 V. d) Line profiles of the AFM images at the location indicated in (a)–(c) for various conditions and gate bias voltages.

To further evaluate the volumetric characteristics of capacitive coupling in CuPc electrolyte‐gated transistors, we have conducted Impedance analysis on CuPc films with varying mass per area, from 15 to 60 µg cm^−^^2^, interfaced with the same agar‐agar hydrogel employed in the transistors of this study (**Figure** **5**a,b).^[^ ^75^ ^]^ All measurements were taken reproducing the full accumulation of the transistor channel (all the details related to EIS measurements are reported in SI and the evolution of EIS spectra with applied bias is reported in Figure S10, Supporting Information). All spectra display a high‐frequency region in the interval between 1 kHz and 1 MHz, marked by a resistive behavior that reflects the bulk resistance of the employed electrolyte (mean value ~150 Ω). In the interval between 100 mHz and 1 kHz, instead, a non‐ideal capacitive behavior (impedance phase within 65° and 80°) is observed and can be attributed to semiconductor/electrolyte interfacial phenomena.^[^ ^75^, ^76^ ^]^ Within this region, the impedance modulus decreases with increasing CuPc mass per area, remarking the volumetric nature of CuPc/electrolyte capacitive coupling. By fitting the impedance spectra with the standard Randles circuit (fitting curves reported in Figure S11, Supporting Information), the electrical double‐layer capacitances (*C*_EDL_) can be extracted, as reported in the plot of Figure 5c. The corresponding Faradaic charge transfer resistance values (*R*_CT_) are reported in Table S1 (Supporting Information).^[^ ^77^ ^]^ The extracted *R*_CT_ values lie in the MΩ cm^−1^ range, indicating negligible Faradaic activity within the OECT operational voltage range. Regarding *C*_EDL_, it increases with increasing film mass per unit area, ranging from a few tens of µF∙cm^−^^2^ for 15 µg cm^−^^2^ films to ≈150 µF cm^−^^2^ for 60 µg cm^−^^2^ films, (corresponding to a volumetric capacitance of ≈4 F cm^−3^). This trend aligns with the observed improvement in *On* currents in transistor architectures (Figure 4b) and highlights the superior capacitive coupling of thicker CuPc films with the electrolyte, consistent with ionic permeation – the key feature of OECTs working mechanism.^[^ ^75^ ^]^ The observed decrease in channel current beyond 60 µg cm^−^^2^ is attributed to the saturation of the Electrical Double Layer capacitance, as confirmed by impedance measurements in Figure S12 (Supporting Information). This saturation, combined with the increased thickness of the undoped CuPc layer, results in elevated contact resistance due to limited ion penetration and charge carrier transport, ultimately leading to reduced device performance.^[^ ^78^ ^]^ To elucidate the ion permeation mechanism, we performed in‐situ AFM measurements of porous CuPc (≈500 nm thick) on gold electrodes with varying gate biases in PBS electrolyte solution gated with custom Ag/AgCl electrode (see **Figure** **6** and Methods section for more details). We did not observe significant passive swelling (Figure 6a,b) nor active swelling upon gate bias up to *V*_GS_ = −1 V (Figure 6c,d). The absence of swelling along with the unaltered fibrous features implies robust structural integrity and stability of CuPc microstructure during the ion permeation process. Such *in‐operando* evidence is consistent with the manifestation of a volumetric capacitance in the form of an extended EDL resulting from the permeation of the electrolyte through the microporosity of the solution‐processed CuPc layer.

## 5 Fully Edible CuPc OECTs

**Figure 7:**
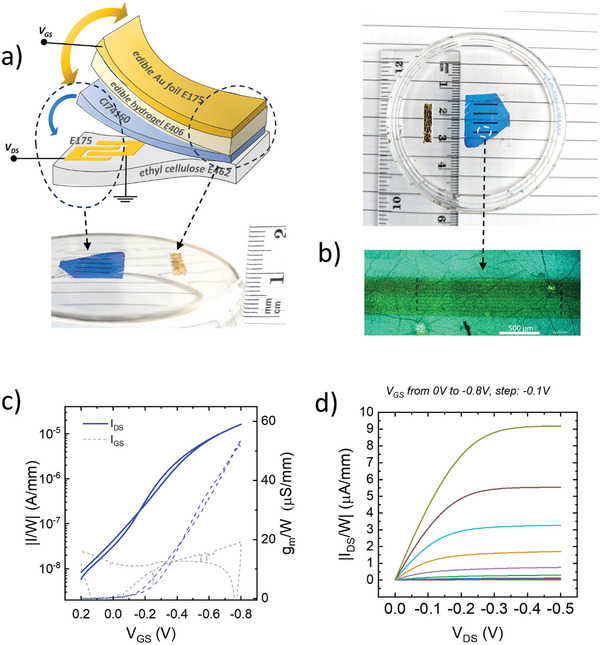
Schematic illustration and photographic pictures of the CuPc‐based edible OECTs on ethyl cellulose a); the edible, laminable, and removable hydrogel consists of an agar‐agar layer imbibed with water/NaCl 0.1 m solutions; optical micrography of the CuPc channel on top of Source and Drain Au electrodes on ethyl cellulose with channel length *L* of ≈5 µm and channel with *W* of ≈20 000 µm b); transfer characteristic curve, transconductance (*g*_m_) versus *V*_GS_ plot (c) and output curve (d) of a 800 nm thick CuPc‐based OECT on ethyl cellulose, gated with 0.1 m NaCl.

OECTs made only with edible materials were fabricated using the proposed strategy for CuPc deposition and the same hydrogel electrolyte and edible gold Gate electrodes. The whole stack was deposited on top of an ethyl‐cellulose substrate with patterned Source and Drain gold electrodes (**Figure** **7**a,b).^[^ ^11^ ^]^ *P‐*type current modulation with ideal ohmic charge injection is clearly observed (Figure 7c,d), with *On/Off* ratio ≈10^3^, *On* current up to 20 µA mm^−1^ at *V_GS_* = −0.8 V, and *V*_th_ ≈ −0.2 V. A common figure of merit to quantify the modulation or amplification efficiency of a transistor is the transconductance (*g_m_*), defined as $g_{m}=\frac{\partial I_{DS}}{\partial V_{GS}}$. We observed transconductance values up to ≈50 µS mm^−1^ (Figure 7c) at *V_GS_* = 0.8 V, exceeding by more than two orders of magnitude the transconductance reported for edible EGOFETs fabricated with evaporated CuPc.^[^ ^30^ ^]^ The averaged transfer characteristic curve and the relative point by point standard deviation over 10 samples, reported in Figure S13 (Supporting Information), highlights the good reliability of the proposed fabrication process even on edible substrates. A 1‐month shelf‐life stability test (T ≈25 °C and relative humidity between 40% and 50%) demonstrates the stability of the fully edible OECTs developed in this work, consistent with the findings of Feltri et al. on thermally evaporated CuPc‐based fully edible EGOFETs (Figure S14, Supporting Information).^[^ ^30^ ^]^

## 6 Electrical Characterization of TFA‐Processed CuPc Films with Variable Purity Levels

**Figure 8:**
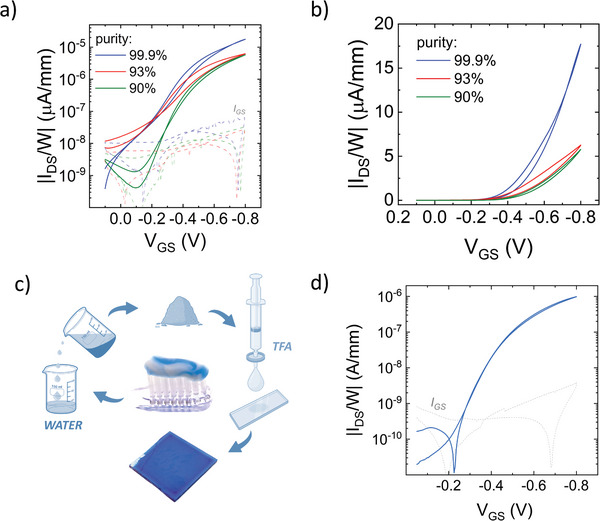
Transfer characteristic curves of optimized CuPc OECTs with variable purity, plot in logarithmic a) and linear b) scales; c) sketch of CuPc extraction procedure from toothpaste; d) representative transfer characteristic curve of OECTs where CuPc was extracted from toothpaste.

The purity of pigments is widely recognized as a crucial factor influencing the transport properties of the corresponding thin films.^[^ ^79^ ^]^ For organic semiconductors, purities of 99% or greater are typically sought after,^[^ ^79^ ^]^ with the purification process posing challenges in terms of complexity and significantly influencing the final cost per gram.^[^ ^79^ ^]^ So far, we have illustrated the transport properties of electronic‐grade, 99.9% pure, CuPc. **Figure** **8**a,b and Figure S15 (Supporting Information) display the comparison of OECTs with CuPc films from commercial batches with different degrees of purity and cost, namely 99.95% (triple‐sublimed grade from Merck, specifically purified for organic electronics application, ≈600 € g^−1^), 93% (β phase from TCI, widely used in printing inks, textiles, and paints due to its superior color strength and brightness, 1.36 € g^−1^) and 90% (α phase from TCI, largely employed in coating and plastics, 1.36 €∙g^−1^).^[^ ^80^ ^]^ Despite a three‐fold reduction of the *On* current (*V*_GS_ = −0.8 V), consistent across the two impure pigments, a discernible and effective charge modulation remains evident, with the *On/Off* ratio largely exceeding 10^2^. This is in stark contrast to the recent results by Irimia‐Vladu and coworkers on solid‐state OFETs obtained using CuPc β phase with 90% purity.^[^ ^79^ ^]^ They measured poor current levels and extracted mobility not superior to few10^−5^ cm^2^ V^−1^ s^−1^ using the untreated powder, requiring three sublimation steps to reach reasonable mobility values in the range of 10^−3^ cm^2^ V^−1^ s^−1^.^[^ ^79^, ^81^ ^]^ To further validate the feasibility of utilizing non‐electronic‐grade pigments, potentially sourced from industrial waste streams such as cosmetics supply chain, we conducted a preliminary extraction of CuPc from a commercially available toothpaste. It should be noted that CuPc is incorporated into toothpaste formulations using commercially available CuPc crystal dispersions in water/glycerin solutions, constituting ≈38% of the formulation. These dispersions (marketed as COVARINE BLUE and Cosmenyl Blue A4R) also contain various wetting agents and surfactants, such as Sodium Lauryl Sulfate, Dimethicone, Phenoxyethanol, Iodopropynyl Butylcarbamate, and Dipropylene Glycol. As illustrated in Figure 8c and described in detail in the Methods section, the extraction process involves two main steps: the separation of all non‐water‐soluble components of the toothpaste, including CuPc and silica, using decantation, followed by a dissolution of all non‐water‐soluble components in TFA and a subsequent filtration step. The resulting CuPc solution was then directly casted on glass, and the resulting films were tested as the active material in OECTs with the architecture depicted in Figure 4a. An exemplary OECT transfer curve is reported in Figure 8d: distinct *p‐*type modulation can be observed, along with substantial current modulation with *On/Off* ratio reaching 10^3^ and, notably, negligible hysteresis. This demonstrates that a promising tolerance to chemical impurities can be achieved using the here proposed processing strategy, paving the way for future exploitation of CuPc upcycling.

## 7 Conclusion

In this study, we have reported the first demonstration of edible OECTs suitable for the in‐body application, utilizing ethyl cellulose with patterned gold as the substrates, edible hydrogels as the gating media,^[^ ^1^, ^82^, ^83^, ^84^, ^85^ ^]^ and CuPc as the ion permeable edible organic semiconductor. CuPc is recognized as one of the most crucial and cost‐effective blue colorants currently available in the market, in virtue of excellent color stability, adhesion properties, resistance to fading, weather resistance and ability to withstand high temperatures and UV exposure.^[^ ^80^ ^]^ In 2010, it was estimated that 21 thousand metric tons of CuPc pigments were used in Europe, inside printing inks, paints, coatings, and plastics.^[^ ^86^ ^]^ Despite not being biodegradable, no phytotoxic effects have been evidenced for CuPc, which, together with a negligible bioavailability, profiles it as a relatively low‐impact material.^[^ ^86^, ^87^, ^88^ ^]^ Additionally, different efforts are ongoing aimed at making CuPc production sustainable, including novel and greener synthetic approaches^[^ ^89^, ^90^ ^]^ and strategies for the treatment of pigment‐containing industrial wastewater.^[^ ^91^ ^]^ CuPc has been just recently proposed for edible electronics applications by Feltri et al.,^[^ ^30^ ^]^ who demonstrated that a daily ingestion of up to 1 mg of CuPc is deemed safe for oral administration. Nevertheless, efficient ion permeability has not been observed using standard deposition methods,^[^ ^30^, ^92^ ^]^ and therefore, CuPc has never been considered for OECTs. In contrast, we have demonstrated a novel deposition strategy based on solvent‐induced molecular protonation, which results in a distinctive fibrous and electrolyte‐permeable film texture. An easy *float‐off and pick‐up* transfer method is then employed to transfer the films onto edible substrates, thereby overcoming the incompatibility of edible substrates, such as the ethyl cellulose used in this study, with the solvents involved in the aforementioned process. We successfully demonstrated the reliable transfer of films ranging from 7.5 to 75 µg mm^−2^. OECTs fabricated using this method achieved optimized performance with CuPc films with a mass per unit area of 60 µg mm^−2^, corresponding to a maximum film thickness of ≈800 nm. For these films, capacitance values up to 150 µF cm^−2^ were demonstrated, i.e., 30 times higher than those of compact CuPc thin films in similar edible electrolyte‐gated configuration,^[^ ^30^ ^]^ and, in general, exceeding by more than one order of magnitude those of standard EGOFETs. As a consequence, efficient transconductance up to ≈50 µS mm^−1^ (normalized to the channel width) and current modulation spanning three orders of magnitude were finally achieved. The best OECTs with the recipe proposed in this study utilize 6 µg of CuPc. Based on the recent quantification of safely ingested CuPc,^[^ ^30^ ^]^ it is extrapolated that the proposed recipe could safely accommodate over 150 CuPc‐based OECTs within an electronic device intended for consumption. In **Table** **1**, the estimated amounts of all the materials constituting a single edible OECT are reported, along with each corresponding Acceptable Daily Intake (ADI) value, in support of the total edibility of the proposed device. We have conducted extensive structural and electrical investigations on CuPc to clarify the non‐trivial ion permeation phenomena occurring through the solution‐processed CuPc films, which underline the functionality of our OECTs. We ultimately attributed them to the porous microstructure that arises from the distinctive fiber‐bundles arrangement in the CuPc film. Specifically, we attribute the observed increase in volumetric capacitance to the formation of a diffuse EDL along the extended interfacial regions formed by electrolyte channeling through the film porous structure. As the mass per unit area increases, the film porosity allows for a greater surface area to be effectively exposed to the electrolyte. To support this statement, we have also realized and characterized solution‐processed CuPc films displaying similar fibrous domains but arranged within a compact meso‐structure (see details in SI Annex A). For these films, no scaling of the capacitive coupling with the volume of CuPc was observed, and the *C*_EDL_ remained invariant at ≈5 µF cm^−^^2^, consistently with standard planar capacitive coupling at the electrolyte/semiconductor interface.^[^ ^30^ ^]^ Therefore, we can conclude that CuPc porosity is a necessary requirement to permit electrolyte permeation and ion conduction through the CuPc films. A porous mesostructure represents an optimal scenario for OECTs operation, as it may resolve the trade‐off between inducing volumetric ionic capacitance and limiting the ion‐induced microstructural swelling affecting the transport in organic semiconductors, as demonstrated through in‐situ AFM of operating CuPc reported in Figure 6.^[^ ^93^, ^94^, ^95^ ^]^ However, to bridge the remaining gap with state‐of‐the‐art non‐Fedible OECTs, generally exhibiting volumetric capacitances at least one order of magnitude higher than our CuPc films,^[^ ^96^, ^97^, ^98^, ^99^ ^]^ further optimization of film porosity is required. This advancement necessitates a more comprehensive understanding and precise parameterization of CuPc self‐assembly mechanisms. Future investigations in this direction could lead to enhanced electrochemical performance of solution processed CuPc films, thereby facilitating the development of ingestible electronic circuits and biosensors. Finally, the proposed process demonstrated a reduced impact of CuPc batch impurities on transport performance, even down to 90% purity. This level of purity can be easily and cost‐effectively achieved through synthetic methods and subsequent solvent washing without the need for expensive and energy‐intensive refinement techniques such as sublimation.^[^ ^79^ ^]^ This finding suggests that the current supply chain, which already supports a vast pigment market, could also be utilized for the low‐cost supply of organic semiconductors in the production lines of future organic electronic devices. They also indicate tangible opportunities for the upcycling of pigment potentially recovered from industrial waste, as exemplified by our demonstration of OECTs made with CuPc directly extracted from a CuPc‐containing toothpaste formulation. F.uture efforts will focus on replacing TFA in the protonation/solvation mechanism, advancing toward more sustainable and green chemistry strategies. Overall, this contribution demonstrates significant potential for the development of edible, sustainable, and low‐cost (see the bill of materials in Table S2, Supporting Information) electronic systems, which could have a profound impact on the fields of healthcare and food safety.

**Table 1:** Estimated amounts of materials constituting a single CuPc‐based OECT, with the corresponding reported Acceptable Daily Intake (ADI) and European Food Safety Authority (EFSA) E numbers.

Material	dose per OECT	ADI
CuPc	6.00 µg	N.A.^*^
Ethyl cellulose	0.05 mg	660÷900 mg kg^−1^ day^−1^ (E462)
Gold	4.00 µg	1.32 µg kg^−1 ^day^−1^ (E175)
Agar‐Agar	0.10 mg	not specified (E 406)
Glycerol	0.50 mg	2 g kg^−1^ day^−1^ (E422)

* Never regulated for ingestion. It is reported in literature that up to 1 mg day^−1^ is safely ingested through commercial toothpaste formulations.^[^ ^30^ ^]^

## 8 Experimental Section

### Materials

Copper(II) phthalocyanine powder (99.9% purity), ethyl cellulose powder (48.0‐49.5% (w/w) ethoxyl basis), Agar powder, ethanol, glycerol, and trifluoroacetic acid were purchased from Merck Life Science. Copper(II) phthalocyanine powder α‐form (90% purity) and β‐form (93% purity) were purchased from TCI. Edible gold foils were purchased from Giusto Manetti Battiloro.

### Extraction Protocol of CuPc from Colgate Toothpaste

To prepare the sample for analysis, the process was initiated by pouring 80 mL of toothpaste into a glass beaker with a volumetric capacity of 1000 mL. This 80 mL of toothpaste was combined with 800 mL of deionized water, resulting in a total volume of 880 mL. To facilitate thorough mixing, a stirring rod or magnetic stirrer could be utilized, ensuring the uniform dispersion of toothpaste within the aqueous medium. The prepared solution was immersed in an ultrasonic bath for a duration of 10–15 s. This step was crucial for breaking down any aggregates or ensuring the dispersion of particulate matter within the solution. The solution was allowed to undergo the process of sedimentation by leaving it undisturbed for a short time (≈1 min). Subsequently, decanting was performed, carefully removing the supernatant liquid while leaving the sediment behind. This sedimentation and decanting process was repeated a total of 5 times to ensure the isolation of the desired substance. the sedimented material was extracted using a pipette, transferring it meticulously into a glass Petri dish. Once the sediment was gathered, the drying process was initiated by placing the Petri dish in an oven set at 60 °C for a duration of 1 h or until complete dryness was achieved. Upon completion of the drying phase, carefully the dried particles were poured on A4 paper to remove any residual small white particles (white particles would be captured on the rough paper surface, while bigger blue particles would roll when the paper was angled). Finally, the dried blue beads were transferred into an Eppendorf tube.

### Devices Fabrication

All CuPc films were deposited as described here. CuPc was dissolved in trifluoroacetic acid (TFA, 1.5 g L^−1^), stirred for 40 min, and then drop‐cast onto microscope slides (usually 4 cm^2^ areas). Variable drop volumes in the 10 µL ÷ 200 µL range were used to adjust the final film mass/thickness. To obtain porous films, the wet films were allowed to dry spontaneously in air under static conditions. After drying, all films were soaked in an ethanol bath for more than 15 min to remove residual traces of TFA. Films were then transferred on different test substrates following the procedure illustrated in Section 6.

Bottom‐gate OFETs were fabricated by transferring CuPc films on top of back‐gated interdigitated substrates with Au source/drain electrodes, 230 nm thick SiO_2_ gate‐insulator, *p‐*doped silicon as the back gate and Au source and drain electrodes with varied channel width and channel length ratio *W*/*L*, from 500 to 4000, purchased from Fraunhofer IPMS. Top‐gate OFETs were fabricated by transferring CuPc films on top of electronic grade Corning glass, gold patterned substrates. Prior to CuPc deposition, all substrates were ultrasonically cleaned in acetone, isopropanol alcohol, and deionized water for 5 min and then cleaned in O_2_ plasma for 5 min. Au source/drain electrodes (*W/L* from 500 to 4000) were defined using photolithography. As for the dielectric, a layer of Poly[4,5‐difluoro‐2,2‐bis(trifluoromethyl)‐1,3‐dioxole‐co‐tetrafluoroethylene] (PTFE), 600 nm thick, was deposited by spin coating a 60 mg mL^−1^ solution in Fluorinert FC‐40. The top gate electrode was obtained by thermal evaporation of 40 nm of aluminum on top of the PTFE film.

The OECTs were fabricated by transferring CuPc films on top of electronic grade Corning glass substrates identical to those used for Top‐gate OFETs. Agar hydrogels were obtained by casting in a Petri Dish kept at room temperature a hot water solution (100 s °C) of agar (40 g L^−1^) and glycerol (8 g L^−1^) with 0.1 m salt concentration (alternatively NaCl and Na_2_SO_4_). After formation, a small piece of agar hydrogel was separated and manually placed on top of the CuPc channel of the OECTs. A piece of gold foil was adjusted on top of the hydrogel to work as the top gate electrode. The edible electrochemical transistors were obtained by transferring CuPc on top of ethyl cellulose substrates with Au source/drain electrodes, prepared using a proprietary, non‐disclosable process. The hydrogel and the gold gate electrodes were assembled using the procedure described above.

Devices for impedance analysis were fabricated by transferring CuPc films on top of Au working electrodes on glass, deposited using thermal evaporation and a shadow mask. More specifically, electronic‐grade Corning glass substrates were employed after ultrasonication in acetone, isopropanol alcohol, and deionized water for 5 min each, and after cleaning under O_2_ plasma for 5 min. The same hydrogel and gold foil employed in OECTs served as electrolyte and counter electrode in impedance measurements.

### Characterization

The measurements of the electrical characteristics of the devices were performed in air using an Agilent B1500A Semiconductor Parameter Analyzer. The UV–vis spectra were recorded using a double‐beam Perkin Elmer l1050 spectrophotometer in the 500–800 nm range. The AFM topographic images were acquired through an Agilent 5500 atomic force microscope operated in acoustic mode.

*GIWAXS measurements* were performed at the BL11‐NCD‐Sweet beamline at ALBA Synchrotron Radiation Facility in Barcelona (Spain) with a Rayonix WAXS LX255‐HS detector. The incident energy was 12.4 eV and the sample‐to‐detector distance was set at 201.346 mm. The angle of incidence αi was 0.12° and the exposure time was 1 s. 2D‐GIWAXS patterns were corrected as a function of the components of the scattering vector with a Matlab script developed by Aurora Nogales and Edgar Gutiérrez (https://mathworks.com/matlabcentral/fileexchange/71958‐grazing‐incidence‐wide‐angle‐x‐ray‐scattering‐representation). Thin films were fabricated following the same process sing route described above and transferred onto highly doped silicon substrates.

All electrochemical measurements were performed using MultiPalmSens4 potentiostat.

*FTIR analysis* was performed using FTIR Bruker Vertex 70, with an attenuated total reflectance (ATR) module, with diamond crystal (module code: A225/Q Platinum ATR, crystal: Diamond).

The spectrum was acquired with a resolution of 4 cm^−1^ in a spectrum between 4000 and 200 cm^−1^. 150 scans were acquired, and 200 background scans were performed. a phase resolution of 16 and a zero‐filling factor of 2 were set, with the “Blackman‐Harris 3‐Term” apodization function.

### In Situ AFM Measurement with Varying Bias

Measurements were performed on Bruker Dimension Icon XR AFM in PeakForce tapping mode with SCANASYST‐FLUID (Silicon Nitride) cantilevers. The source and drain electrodes were shorted, and the gate voltage was applied using an Agilent B2912A source measuring unit with a custom Ag/AgCl electrode in PBS 1X electrolyte solution during imaging. The Ag/AgCl electrode was prepared by dip‐coating coiled platinum wire in silver/silver chloride (60/40) paste purchased from Sigma‐Aldrich and dried in the oven at 60 °C for 30 min. The prepared Ag/AgCl electrode was conditioned in a separate buffer PBS 1X solution before use. The device was dried and left overnight in the nitrogen glove box before in situ AFM measurements. The gate current was recorded during imaging, which corroborates with the transfer curve measurements. The acquired AFM images were flattened by three‐point levelling using Gwyddion software.

## Conflict of Interest

The authors declare no conflict of interest.

## Author Contributions

A.L. conceived the original idea. A.L. and M.C. designed the research study. F.M.F. acquired and analyzed the EIS data sets. M.B. fabricated the fully edible substrates with patterned Source and Drain (Au). A.L. fabricated all CuPc films. F.M.F. fabricated all the edible hydrogel. A.L., F.M.F., and M.B. performed the electrical characterization of CuPc OECTs. A.L. performed the AFM analysis. A.D.S. performed the data processing and analyses of GIWAXS data set. B.P., S.K., and G.S., designed and executed the extraction procedure of CuPc crystals from the toothpaste. S.F.G. acquired and analyzed the FTIR data sets. S.T. performed in‐situ AFM measurement with varying bias on CuPc OECTs. A.K. performed time response analysis of CuPc OECTs. All authors contributed to the writing of the manuscript. All authors have approved the final version of the manuscript.

## Supporting information



Supporting Information

## Data Availability

The data that support the findings of this study are available from the corresponding author upon reasonable request.
