# Supplementary material for: A Solution‐Based Deposition Method Enabling Pigment Blue Edible Electrochemical Transistors
Source: Adv Sci (Weinh). 2025 Feb 27;12(29):2416141. doi: 10.1002/advs.202416141 (PMC12362739; doi:10.1002/advs.202416141)
Supplement: Supplementary file 1 — Supporting Information [file ADVS-12-2416141-s001.docx]

Supporting Information

**A Solution-based Deposition Method Enabling Pigment Blue Edible Electrochemical Transistors**

*Alessandro Luzio^*^, Fabrizio M. Ferrarese, Matteo Butti, Alberto D. Scaccabarozzi, Bojan Petrović, Sanja Kojic, Simone Fiorini Granieri, Shubham Tanwar, Adrica Kyndiah, Mario Caironi^*^*

A. Luzio, F. M. Ferrarese, M. Butti, A. D. Scaccabarozzi, S. Fiorini Granieri, S. Tanwar, A. Kyndiah, M. Caironi

Center for Nano Science and Technology, Istituto Italiano di Tecnologia, Via Rubattino, 81, Milano, 20134, Italy

E-mail: alessandro.luzio@iit.it, mario.caironi@iit.it

A. D. Scaccabarozzi (current affiliation), F. M. Ferrarese,

Department of Physics, Politecnico di Milano, Piazza Leonardo da Vinci, 32, Milano 20133, Italy

B. Petrović

Faculty of Medicine, University of Novi Sad, Hajduk Veljkova 3, 21000, Novi Sad, Serbia

S. Kojic, G. Stojanović

Faculty of Technical Sciences University of Novi Sad, T. Dositeja Obradovi´ca 6, Novi Sad 21000, Serbia

***
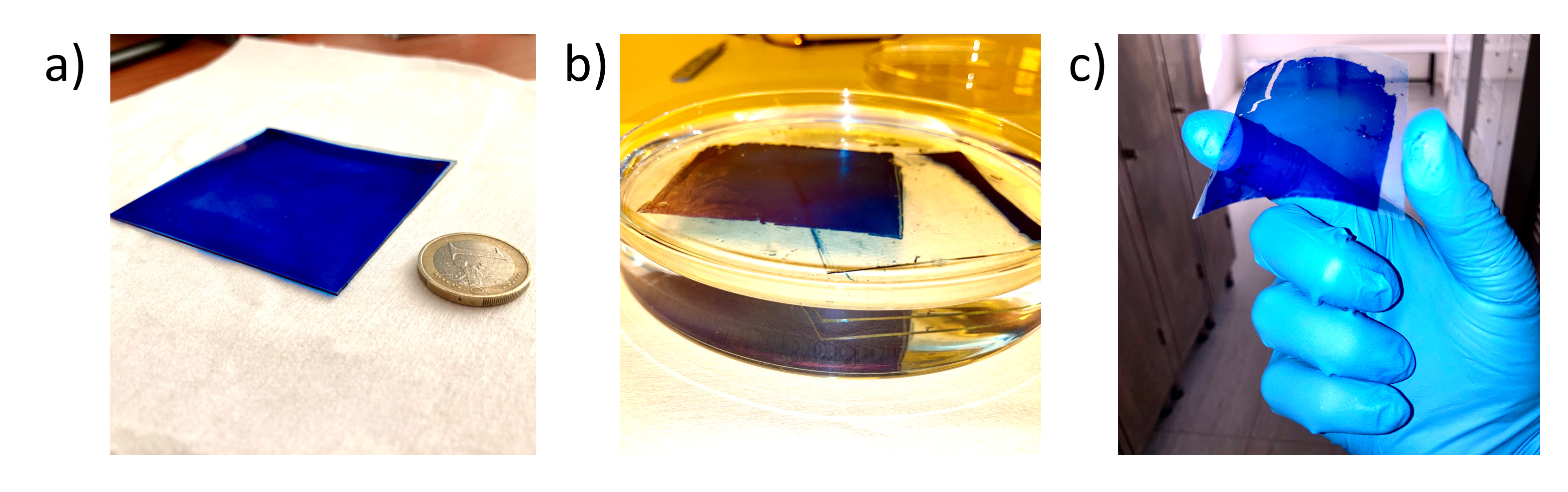
***

**Figure S1** a) picture of the CuPc film drop cast on 7 × 7 cm^2^ glass substrate; b) picture of 7 × 7 cm^2^ CuPc film floating at the water surface; picture of 7 × 7 cm^2^ CuPc film after transferring on top of a PEN flexible substrate.


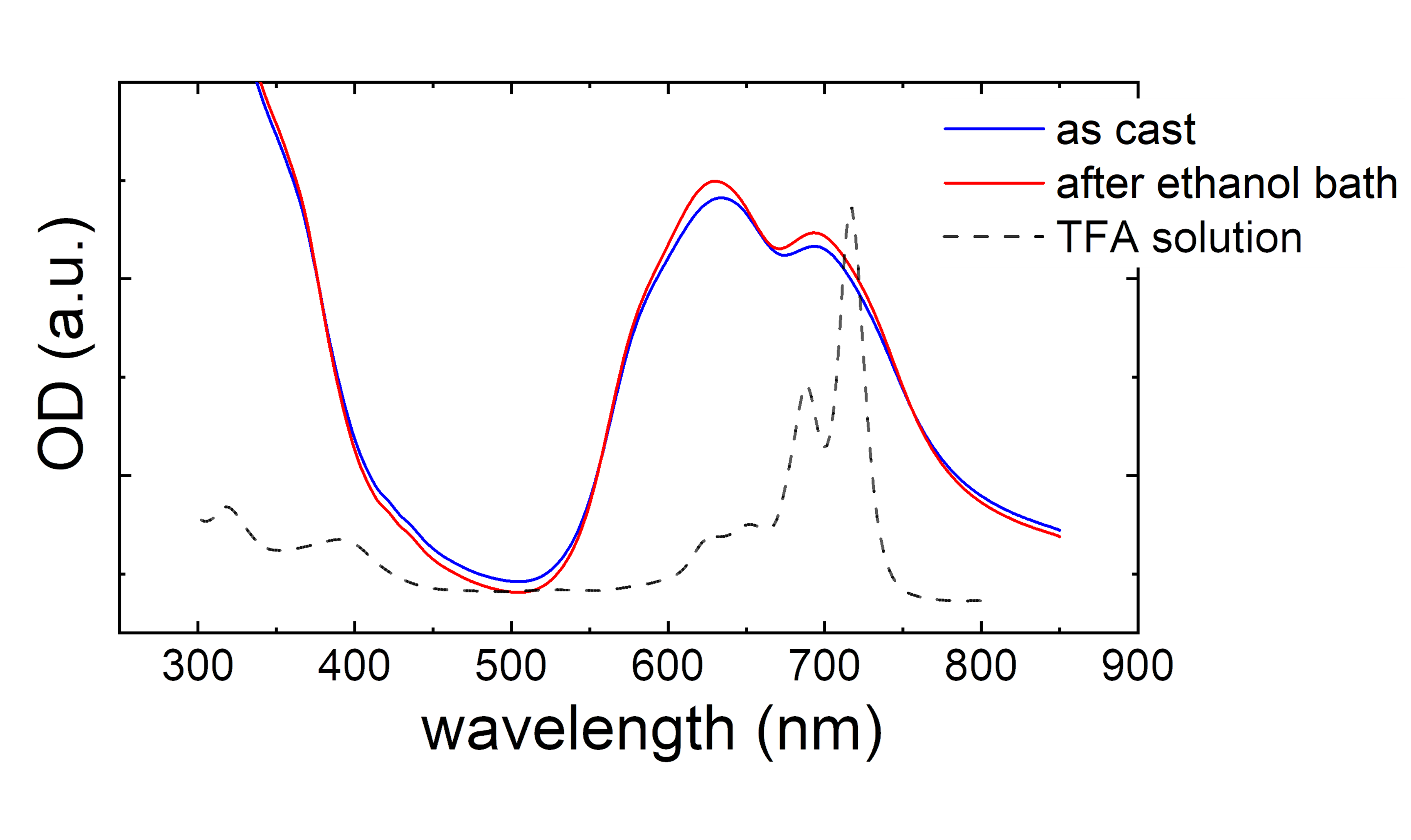


**Figure S2** a) optical density of CuPc films after drop casting (blue line) and after purification through the ethanol bath (red line); the optical density of CuPc in TFA solution (1.5 g/l) is also reported.

Nearly identical spectra are observed before and after the purification process. In both UV-Vis spectra, the Soret and Q bands characteristic of the unsubstituted phthalocyanines can be discerned.^1, 2^ The Soret band peaks at λ ⁓ 300 nm (not shown), while the Q band consists of two main components, peaking at λ ⁓ 650 nm and λ ⁓ 700 nm. The specific shape of the Q band and the relative intensity ratio of its peaks indicate the formation of the well-established CuPc crystalline α-phase.^1^ The crystalline structure of the as-cast films remains unchanged after exposure to the ethanol bath, as evidenced by the unaltered optical absorption characteristics. In the UV-Vis spectrum of CuPc in TFA solution the contribution associated to CuPc2+ are clearly detectable within the 650 nm ÷ 550 nm range.^1^


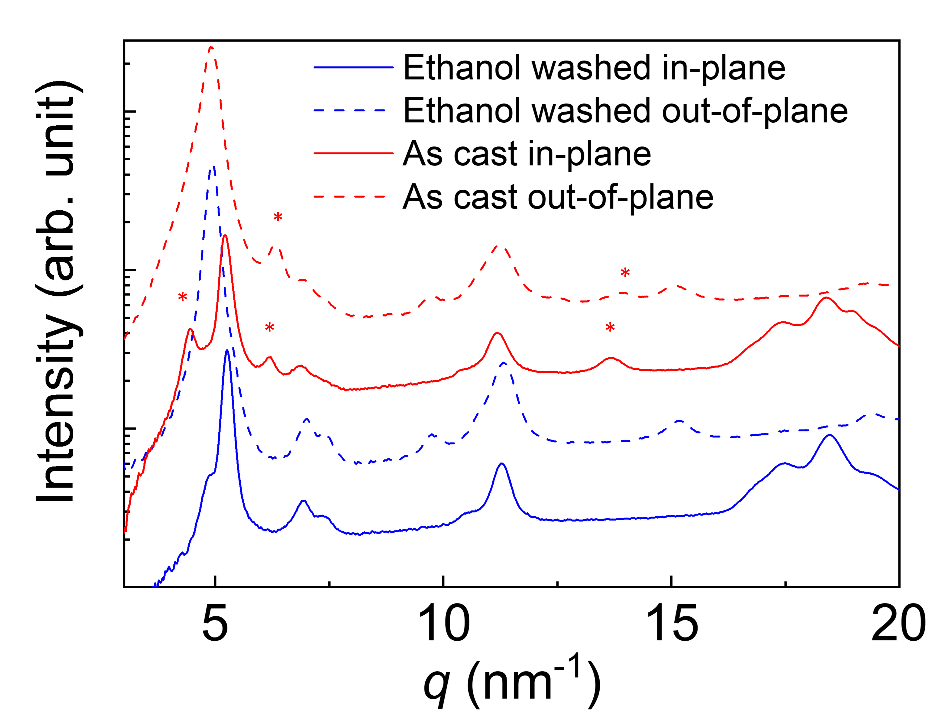


**Figure S3** GIWAXS profiles along the out-of-plane (dashed lines) and in-plane (solid lines) directions for as-cast films (red lines) and further ethanol treated (blue lines) of 1200 nm thick films prior floating.


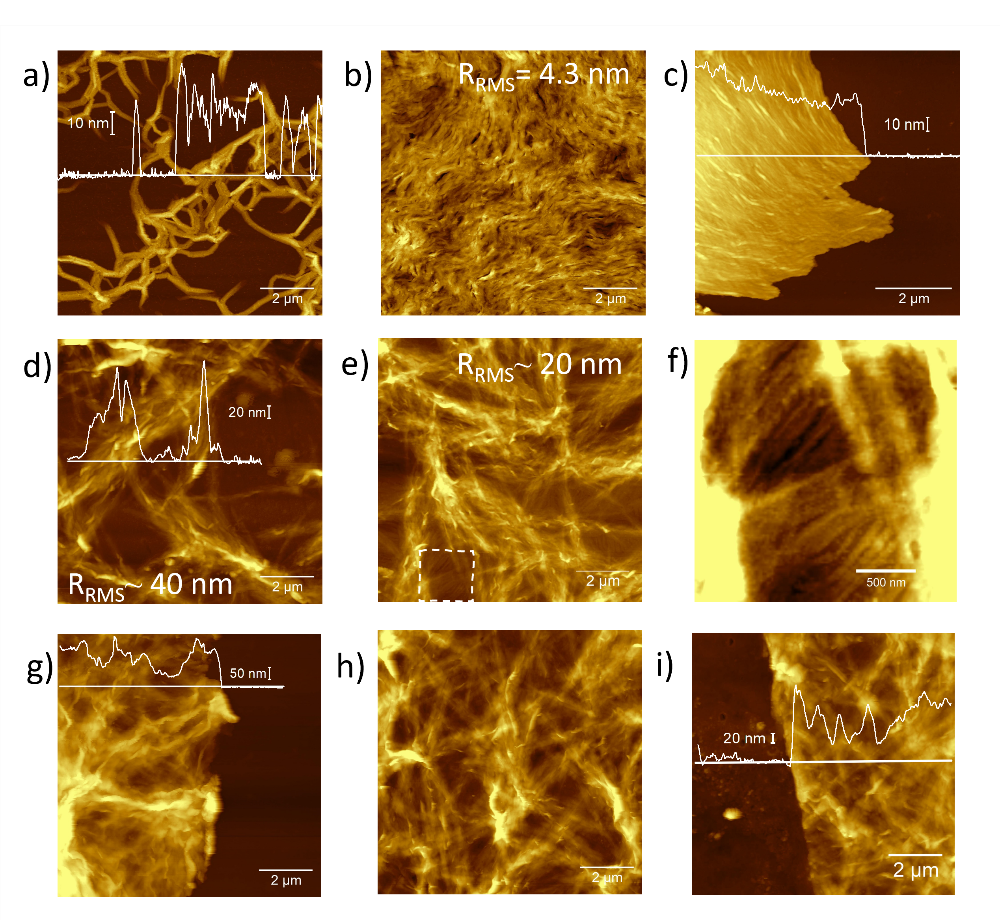


**Figure S4** AFM topographies of CuPc films, obtained by drop casting drops with the following mass per area:: 3 µg cm^-2^ (a), 7.5 µg cm^-2^ (b,c), 15 µg cm^-2^ (d), 30 µg cm^-2^ (e,f,g) (in panel f, a zoom of panel e, to highlight the presence of the CuPc sublayer underneath the bundles), 30 µg cm^-2^ after transferring to ethyl cellulose (h,i)


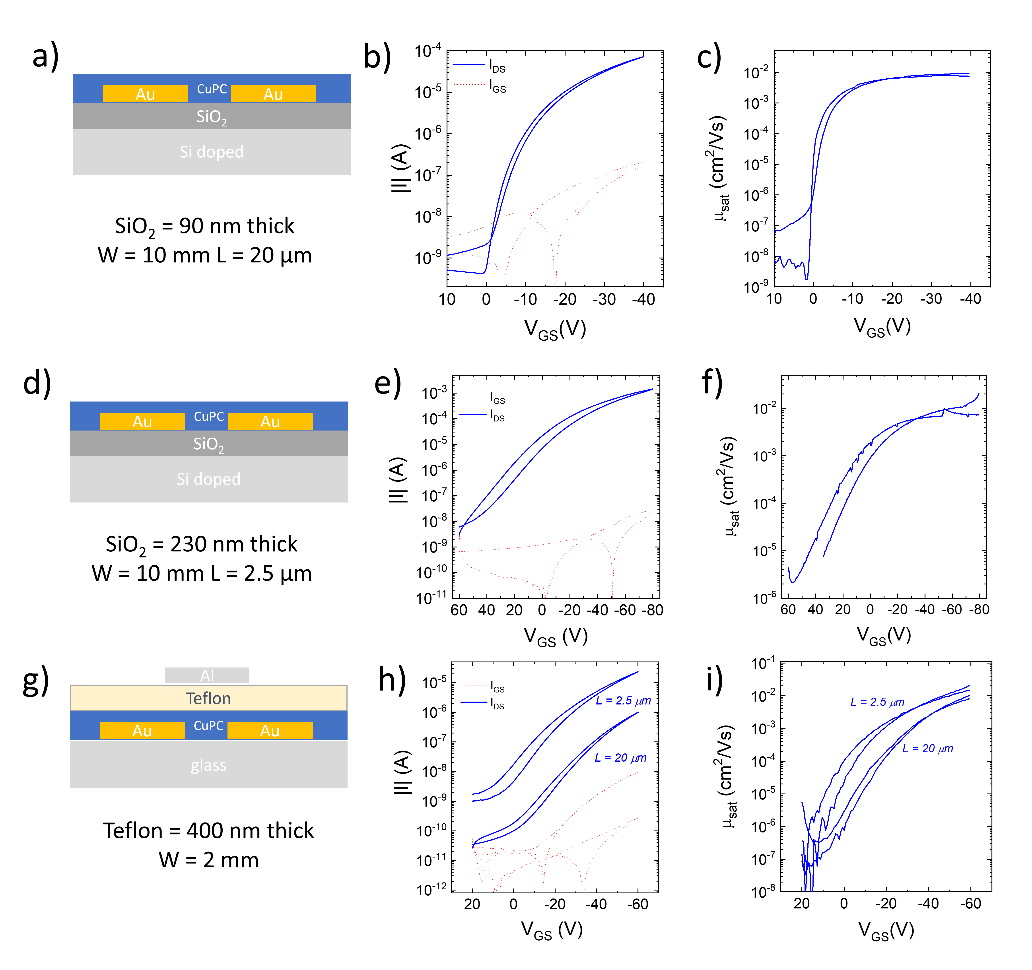


**Figure S5** Transistor structure (a,d,g) and representative transfer characteristic curves (b,e,h) and saturation mobility vs. gate bias V_GS_ plots (c,f,i) of OFETs with CuPc films from TFA solution, ⁓50 nm thick, respectively: in bottom gate, bottom contact configuration with long channel length (a,b,c), bottom gate, bottom contact configuration with short channel length (d,e,f), top gate, bottom contact configuration with long and short channel length (g,h,i). Apparent saturation charge carrier mobility values (in the main text referred as field effect mobility) were extracted by the transfer characteristic curves according to the expression ${I_{DS}=\mu\times C_{die}\times W/2L\times\left( V_{GS}-V_{T} \right)}^{2}$ , where *I_DS_* is the drain current, μ is the saturation mobility, *C_die_* is the specific dielectric capacitance, *W* and *L* are the width and the length of the channel, respectively, *V_GS_* is the gate voltage, *V_DS_* is the drain voltage and *V_T_* is the threshold voltage. Accordingly, the *V_GS_* dependent values of μ were obtained from the slope of *I_DS_^0.5^* vs. *V_GS_*), calculated every three points around each *V_g_* value.^3^


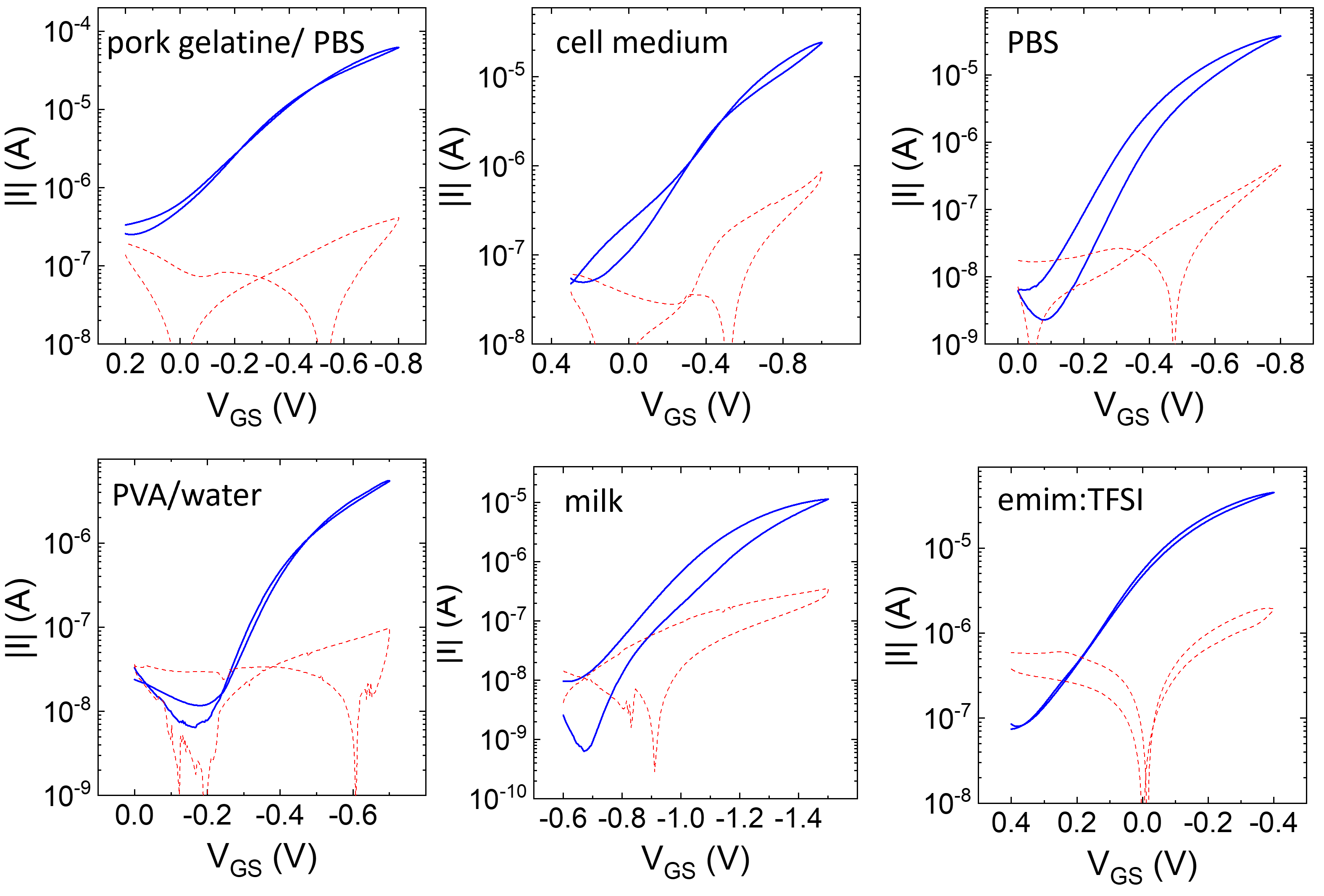


**Figure S6** Transfer characteristic curves of CuPc based transistors gated with various water based biocompatible electrolyte systems and the emim:TFSI ion gel.


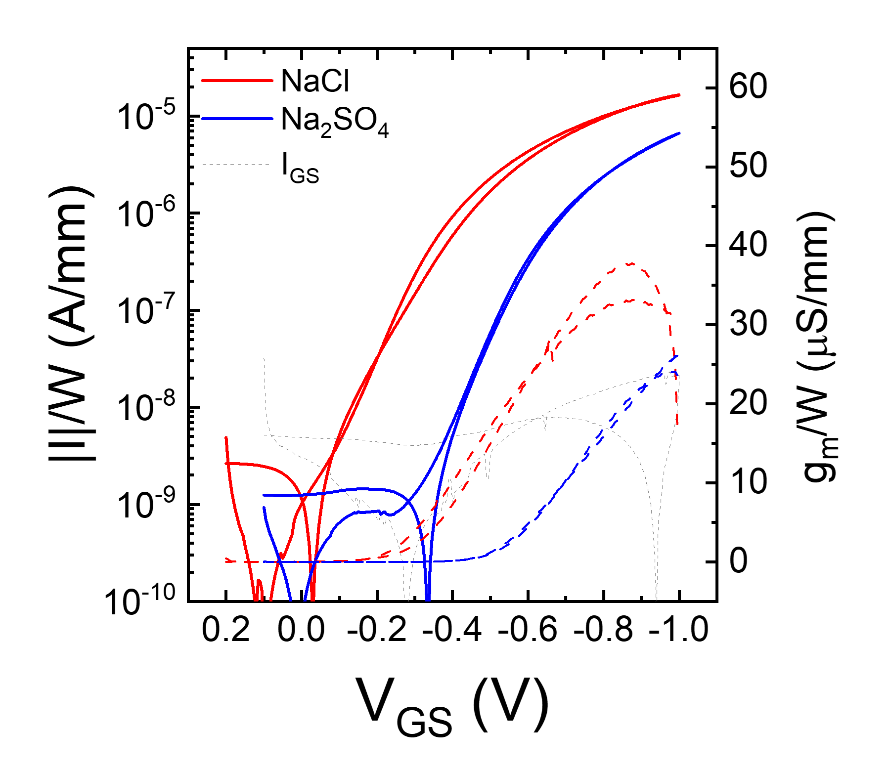


**Figure S7** Transfer characteristic curves of CuPc based OECTs with 60 µg/cm^2^ CuPc mass per area, gated once with Na_2_SO_4_ 1.0M hydrogel once with NaCl 1.0M hydrogel.


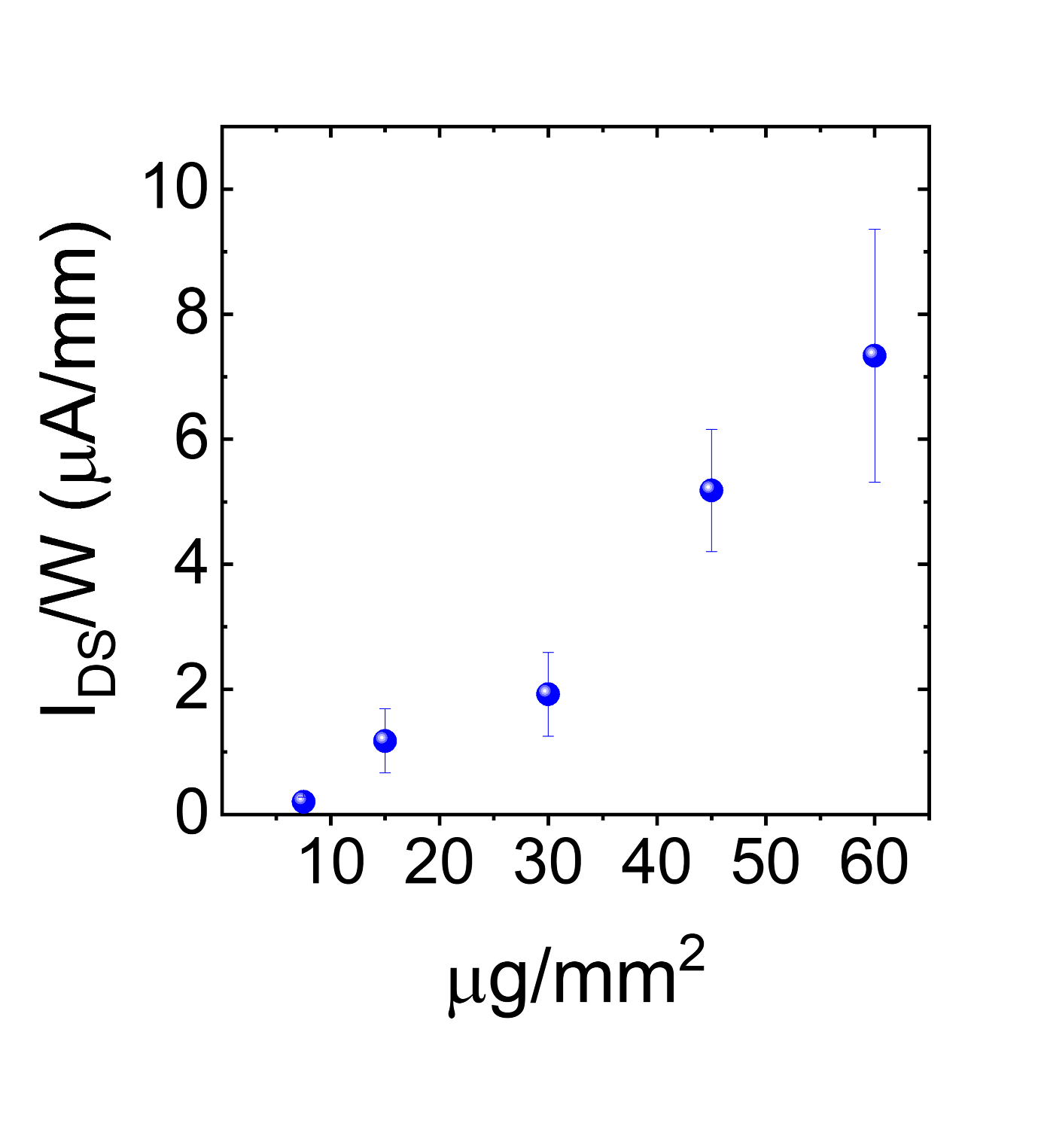


**Figure** **S8** Plot of Source to Drain current normalized to the channel width (I_DS_/W, measured at V_DS_ = -0.5V, V_GS_ = -1V) vs. CuPc mass per unit area


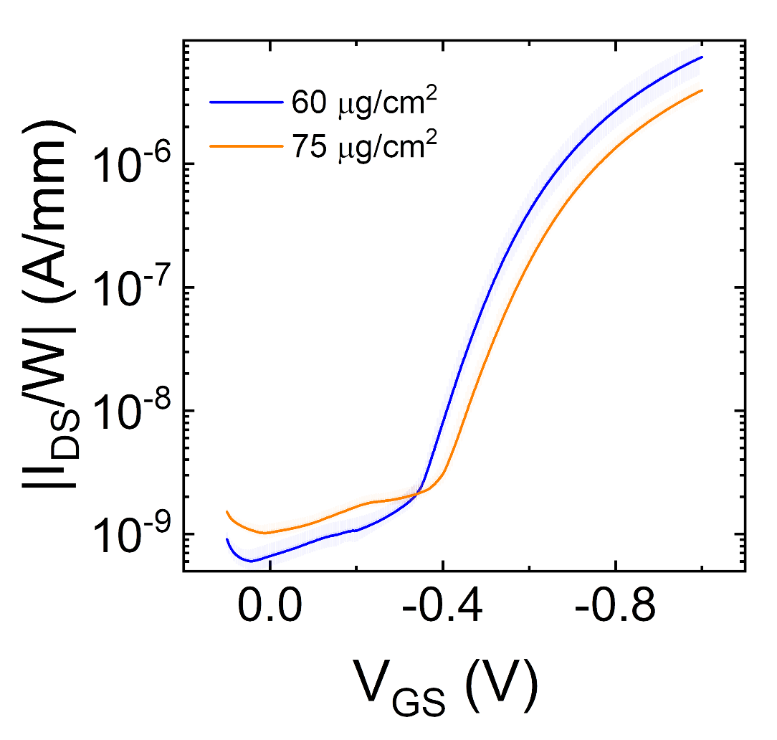


**Figure S9** Mean transfer characteristic curves and point-by-point standard deviation (over 10 measurements) of Na_2_SO_4_-gated CuPc based OECTs with 60 µg/cm^2^ and 75 µg/cm^2^ CuPc mass per area


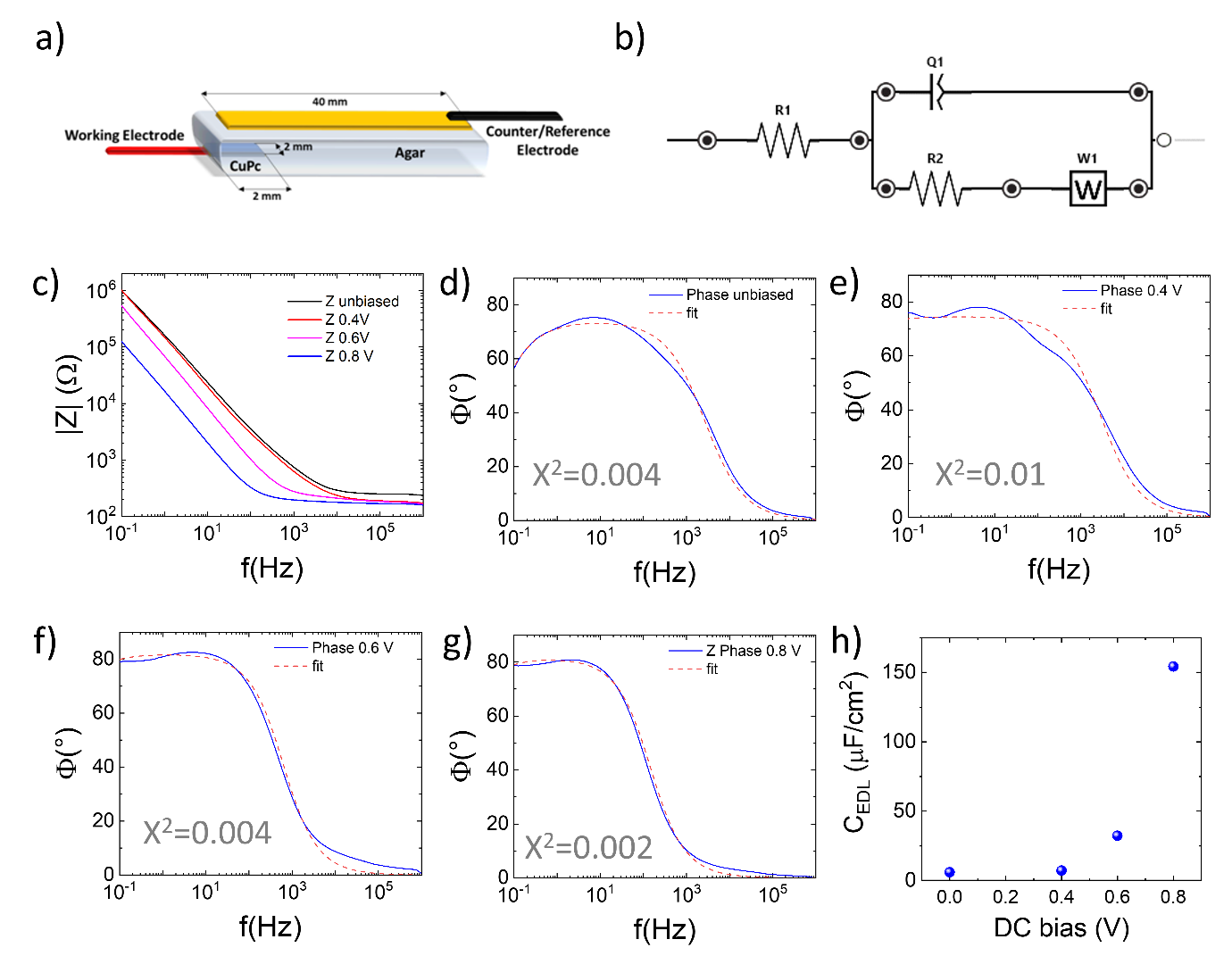


**Figure S10** Scheme of the 2-electrode system architecture employed for EIS measurements of this work (a); scheme of the Randler circuit employed for fitting the EIS measurements of this work (b), where Q1 represents the Constant Phase Element (CPE) and W1 is the Warburg element, eventually added to improve the fitting (a); impedance modulus of EIS measurements on porous CuPc films with mass per area of 60 µg/mm^2^ and variable DC bias, interfaced with NaCl 1M agar hydrogels (c); Randler circuit fitting of impedance phase measurements (d-g); electrical double layer capacitance values C_EDL_ (extracted assuming the Randlar circuital model) vs. DC bias (h).

**
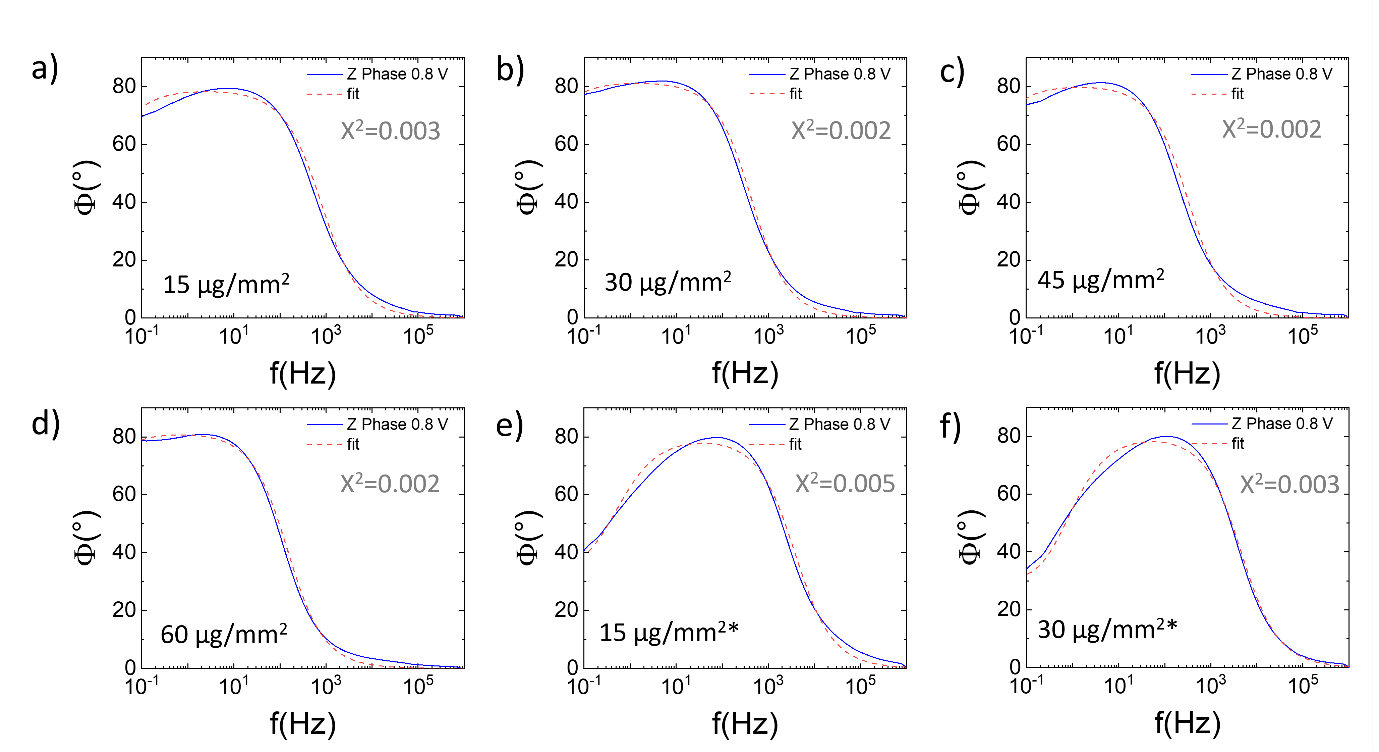
**

**Figure S11** Randler circuit fitting of impedance phase measurements on porous CuPc films with variable mass per area, interfaced with NaCl 1M agar hydrogels.

EIS analysis was carried out with a 2-electrode system (Figure S10a) over a frequency range of 100 mHz to 1 MHz using a 0.1 V AC potential. In addition, an DC potential at 0.8 V was applied on working electrode fabricated with evaporated gold and CuPc semiconductor on top of it in order to simulate the charge accumulation phenomena of the transistors. The Bode plots in Figure S10 and S11 reveal two behaviors: at high frequencies (10 kHz to 1 MHz), the impedance stabilizes around 200 Ohms with phase values near zero, indicating resistive behavior; at low frequencies (≤ 10 kHz), impedance and phase values rise linearly, reaching approximately 80°, suggesting a capacitive behavior due to EDL formation at the electrolye and Cupc semiconductor interface. To model the impedance spectra, a Randles equivalent circuit was employed, where R_bulk_ represents the electrolyte's bulk resistance and R_ct_ indicates the resistance due to charge transfer phenomena at interface. A Constant Phase Element (CPE) was used to modelize the non-ideal capacitive behavior indicated by *T* and that is regulated by the fitting parameter ϕ. For ideal capacitance ϕ values is equal to 1. Finally, the Warburg element represents a frequency-dependent impedance that describes mass diffusion processes. By fitting the data, we can extract the double layer capacitance (C_bottom_) at the bottom electrode when ϕ exceeds 0.75 with the formula:

$$C_{bottom}= T^{\frac{1}{\varphi}}{(\frac{1}{R_{bulk}}+\frac{1}{R_{ct}})}^{1-\frac{1}{\varphi}}$$

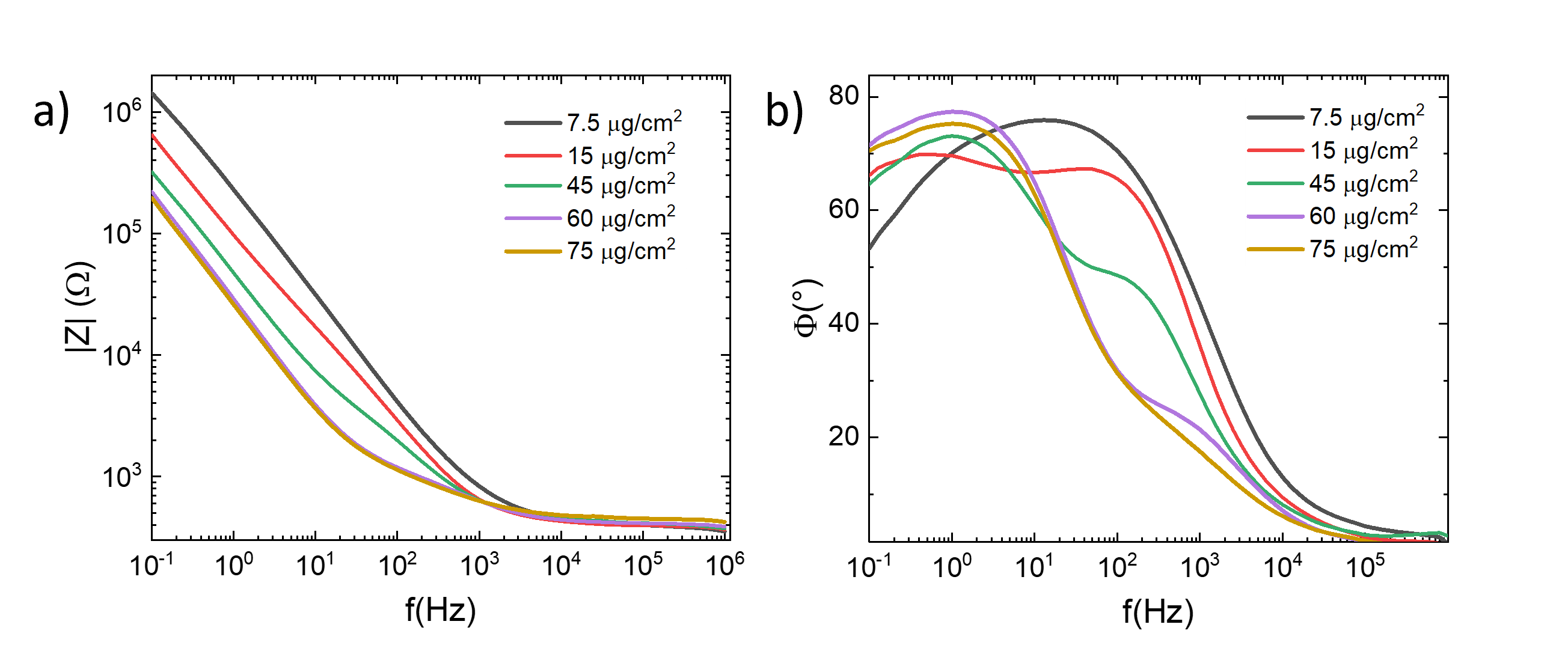


**Figure S12** Bode plots (impedance modulus (a) and phase (b)) of EIS measurements on porous CuPc films with variable thickness interfaced with agar hydrogels including sodium sulphate 1M in water as the electrolyte.

| CuPc mass per area (µg/mm^2^) | T(µF/cm^2^) | φ | Rct(Ohm) |
| --- | --- | --- | --- |
| **15** | 4.321x10^-6^ | 0.877 | 3x10^6^ |
| **30** | 7.495x10^-6^ | 0.904 | 3 x10^6^ |
| **45** | 8.743x10^-6^ | 0.895 | 2.1 x10^6^ |
| **60** | 11.73x10^-6^ | 0.906 | 2.9 x10^6^ |
| **15 compact** | 1.22 x10^-6^ | 0.805 | 7.7 x10^5^ |
| **30 compact** | 1.17 x10^-6^ | 0.848 | 4.8 x10^5^ |

**Table S1** Summary of Randles circuit fitting parameters of impedance spectra of porous CuPc films with variable mass per area, interfaced with NaCl 1M agar hydrogels


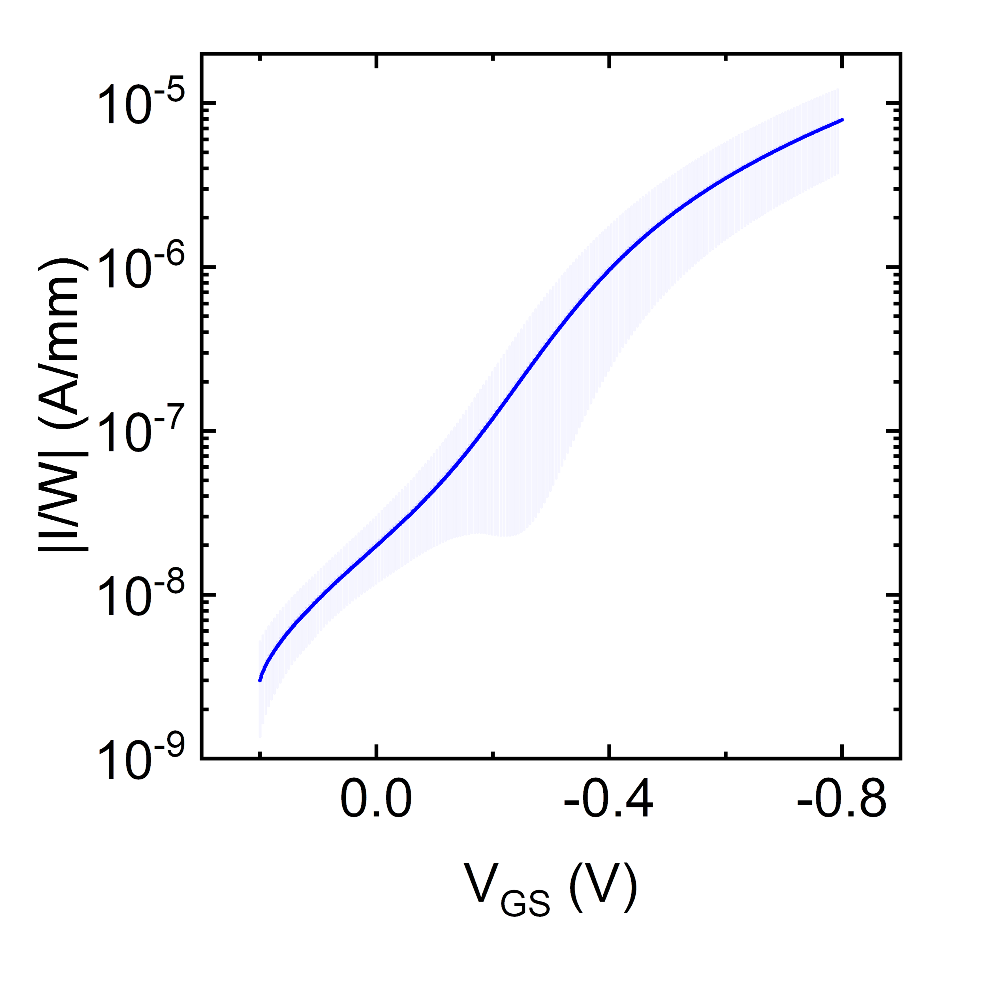


**Figure S13** Mean transfer characteristic curves and point-by-point standard deviation (over 10 measurements for each point) of NaCl-gated fully edible CuPc-based ECTs with 800 nm CuPc thickness.


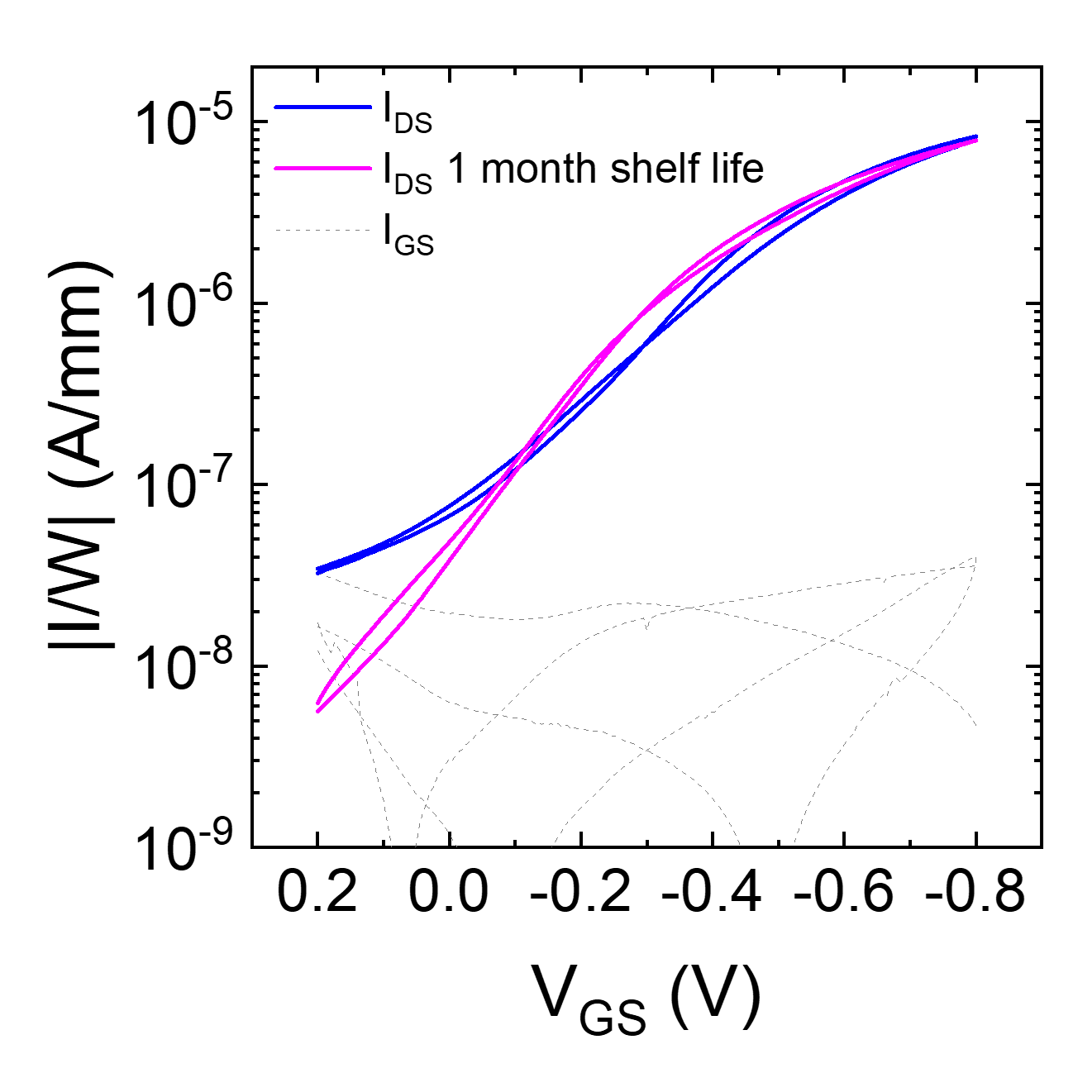


**Figure S14** transfer characteristic curve of CuPc based OECTs (V_DS_ = -0.5V) after fabrication and after 1 month storage in ambient at T ⁓ 25 °C and relative humidity between 40% and 50%.

**Table S2** Bill of materials for a single device: estimated amounts of materials constituting a single CuPc-based OECT, with the corresponding cost and the estimated material cost of a single CuPc-based OECT.

| **Component** | **Cost per gram (€)** | **Quantity used (mg)** | **Cost per device (€)** |
| --- | --- | --- | --- |
| CuPc | 1.36 | 0.006 | 0.000008 |
| Gold electrodes | 60 | 0.004 | 0.00024 |
| Ethyl cellulose | 0.38 | 0.05 | 0.000019 |
| Hydrogel electrolyte | 0.25 | 0.1 | 0.000025 |
| **Total cost** |  |  | **0.000292** |

*
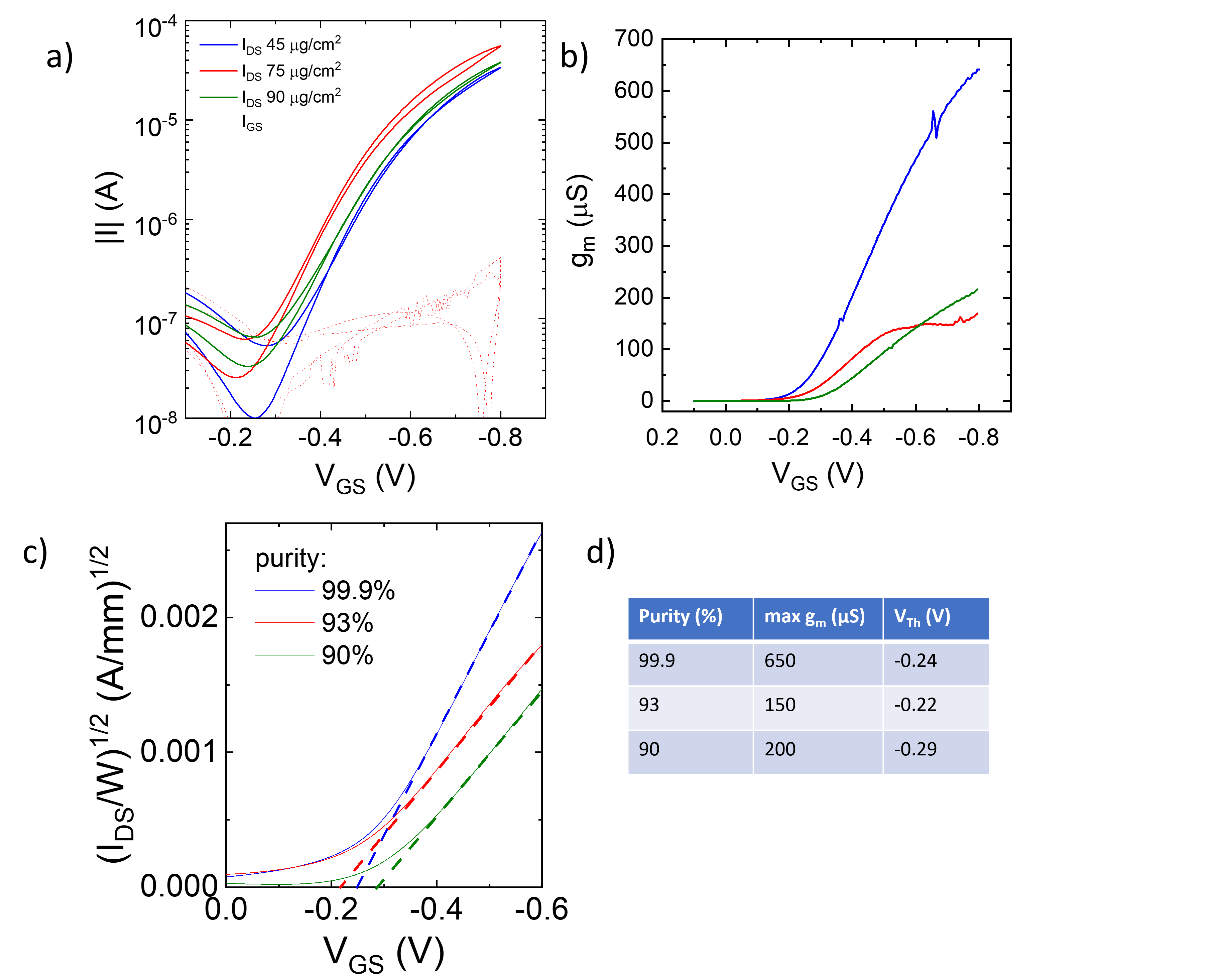
*

**Figure S15** a) transfer characteristic curves (V_DS_=0.5V) of OECTs with CuPc 93% pure, with varying mass per area; transconductance vs. V_GS_ extracted from the transfer characteristic curves of Figure 8a; c) squared root I_DS_ vs V_GS_ extracted from the transfer characteristic curves of Figure 8a; d) summary table of extracted max transconductances and threshold voltages from batches with variable purities.

*Annex A: CuPc compact films from TFA solution.*

Deposition method: CuPc was dissolved in trifluoroacetic acid (TFA, 1.5 g/L), stirred for 40 minutes, and then drop-cast onto microscope slides (usually 4 cm^2^ areas). Compact films were produced by mildly confining the drying environment under a petri dish to create a TFA-saturated atmosphere that did not impede solvent evaporation but slowed considerably. Additionally, the substrate was continuously tilted during drying to facilitate re-dissolution and recrystallization throughout the entire drying process.

**
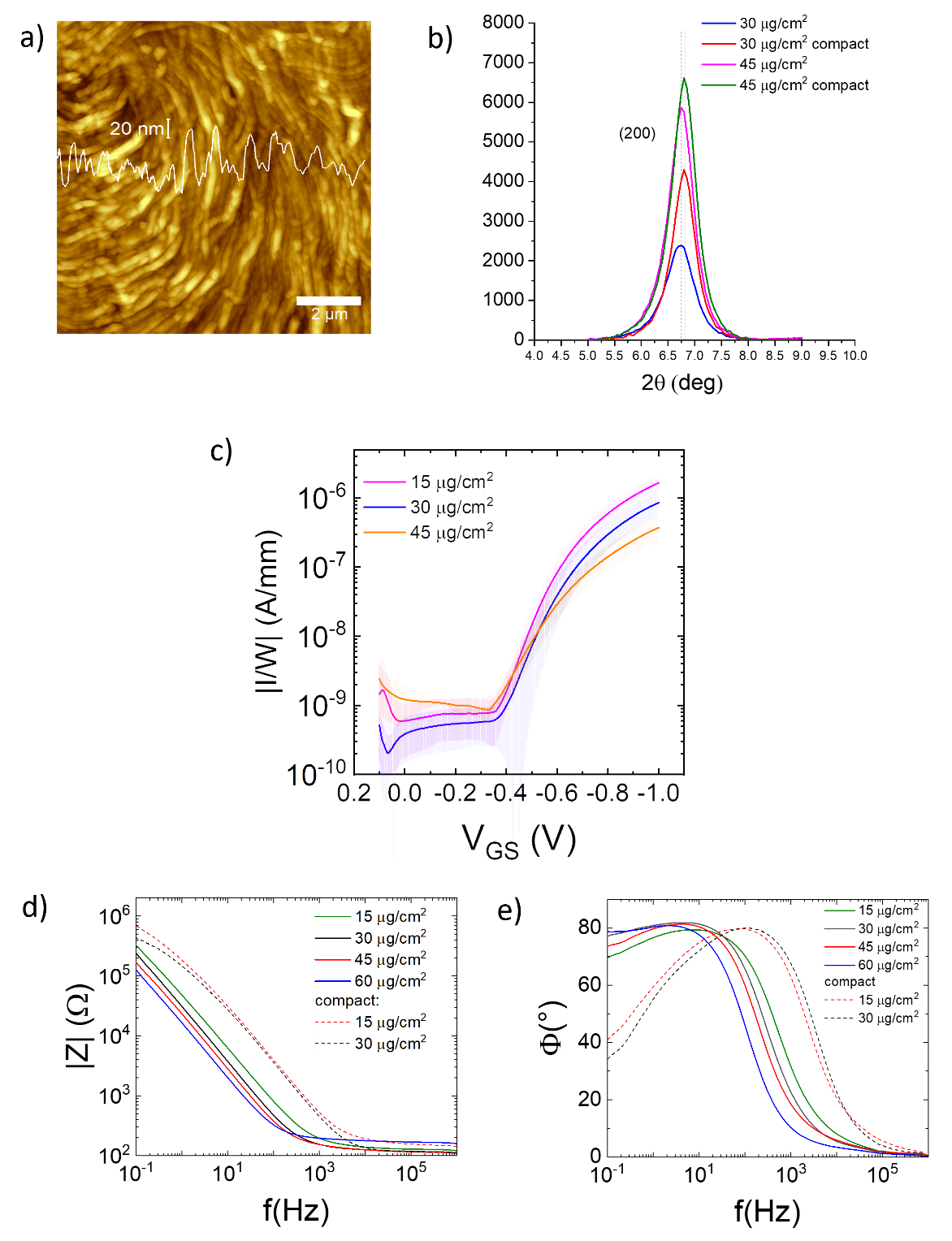
**

**Figure S16** a) AFM topography of CuPc compact films; b) 2D-GIWAXS pattern of TFA processed, porous and compact CuPc films, with variable thickness. A slight shift of the 2θ angle of (200) peak can be observed in case of compact films, likely associated to a slight tilt of the molecular orientation with respect to the substrate. The different peak intensity may be associated with a different local density or to a higher crystallinity of compact films due to the prolonged duration of the crystallization process; c) mean transfer characteristic curves and point by point deviation standard (over 10 measurements) of Na_2_SO_4_-gated OECTs based on compact CuPc with variable mass per area; Bode plots (impedance modulus (d) and phase (e)) of EIS measurements on porous and compact CuPc films with variable thickness interfaced with NaCl 1M agar hydrogels.


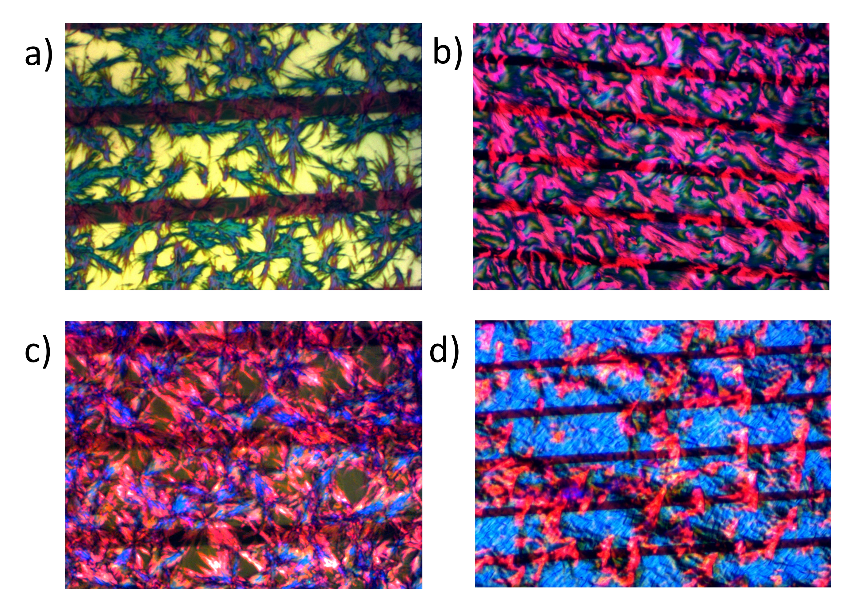


**Figure S17** Polarized light microscopy images (100× magnification, in epi-reflection) topographies of CuPc films transferred on gold patterned glass substrates, obtained by drop casting 40 µl (a,b) and 80 µl (c,d) from a TFA solution with concentration 1.5 g/l on 2 cm^2^ glass surface, once in air and on static substrate (a,c), once in TFA environment and on tilting substrate, to obtain a compact film (b,d).

***References:***

1. Komino, T.; Matsuda, M.; Tajima, H., The fabrication method of unsubstituted planar phthalocyanine thin films by a spin-coating technique. *Thin Solid Films* **2009,** *518* (2), 688-691.

2. Farag, A., Optical absorption studies of copper phthalocyanine thin films. *Optics Laser Technology* **2007,** *39* (4), 728-732.

3. Sze, S.; Ng, K. K., *Physics of semiconductor devices*. 2006; Vol. 3.
